# Hyposaline LUSO Mineral Water Drives Murine Macrophage Polarization Towards an Anti‐Inflammatory M2 Phenotype

**DOI:** 10.1002/fsn3.71962

**Published:** 2026-07-08

**Authors:** Beatriz Rodrigues, Cláudia Cid, Rosa Resende, Mylène Carrascal, Maria do Céu Sousa, Conceição Egas, Cláudia F. Pereira, Marisa Azul, Maria Teresa Cruz, Rui Soares, Ana Silva

**Affiliations:** ^1^ CIBB – Center for Innovative Biomedicine and Biotechnology/CNC – Center for Neuroscience and Cell Biology University of Coimbra Coimbra Portugal; ^2^ Faculty of Pharmacy University of Coimbra Coimbra Portugal; ^3^ CACC – Clinical and Academic Centre of Coimbra Rectory of Universidade of Coimbra Coimbra Portugal; ^4^ Tecnimede Pharmaceutical Companies Group Sintra Portugal; ^5^ Biocant – Transfer Technology Association Biocant Park Cantanhede Portugal; ^6^ Faculty of Medicine University of Coimbra Coimbra Portugal; ^7^ Clinical Pathology Service Portuguese Oncology Institute of Coimbra Coimbra Portugal; ^8^ Luso Thermal SPA Luso Portugal

**Keywords:** anti‐inflammatory, HMOX1, macrophage polarization, natural mineral water, phagocytosis

## Abstract

The beneficial effect of Natural Mineral Water (NMW) in the alleviation and/or treatment of inflammation‐associated diseases has long been empirically recognized, but its scientific validation is still scarce. This study focused on the effect of a Portuguese NMW in modulating the phenotypical characteristics of macrophages, both at homeostasis and in an inflammatory context. As far as we know, we were the first to address the effects of NMW on macrophage polarization. We used RAW 264.7 macrophages cultured in regular medium (control) or LUSO NMW‐containing medium, alone or exposed to lipopolysaccharide (LPS). We explored LUSO NMW effect on macrophage profile (M1, pro‐inflammatory or M2, anti‐inflammatory), release of inflammation mediators, and phagocytic capacity. LUSO NMW‐cultured macrophages acquired an M2 phenotype and showed decreased *Il6* gene expression, and IL‐1β, IL‐6 and tumor necrosis factor‐alpha secretion. Moreover, treated cells exhibited enhanced LPS‐induced phagocytosis. In addition, gene expression and protein levels of the antioxidant enzyme Heme Oxygenase 1 were also increased in LUSO NMW‐exposed cells. Overall, these results strongly support the anti‐inflammatory effect of LUSO NMW and suggest that it may also activate antioxidative pathways, which should be deeply explored, including its use as an adjuvant in immune‐ and anti‐inflammatory‐based therapies.

## Graphical abstract

RAW 264.7 macrophages were cultured in DMEM culture medium made with deionized water (Ctr) or LUSO NMW, bottled (B) and collected directly from the spring (S). To mimic inflammation and induce macrophages' M1 phenotype, the cells were exposed to LPS (100 ng/mL). LPS binds to TLR4 receptors, leading to NF‐kB nuclear translocation, which induces pro‐inflammatory gene transcription (e.g., *Nos2*, *Il1b*, *Il6*, *Tnfa*), increasing the expression and secretion levels of their encoded proteins (e.g., iNOS, IL‐1β, IL‐6, TNF‐α) or their products (such as NO produced by iNOS). Reactive oxygen species (ROS) are also produced in the cytoplasm and mitochondria (mtROS), leading to oxidative stress. ROS further induce nuclear translocation and activation of nuclear factor erythroid 2–related factor 2 (Nrf2), which is responsible for the transcription of antioxidant and detoxifying genes (e.g., *Hmox1*, *Sod2*). In our work, LPS‐activated Ctr macrophages exhibited an increase in CD86 (M1 marker), a decrease in CD206 (M2 marker) expression, and an increase in both CD86/CD206 and CD86/163 ratios. Also, they produced higher levels of pro‐inflammatory mediators (NO, iNOS, IL‐1β, IL‐6 and TNF‐α). Unstimulated macrophages exposed to LUSO NMW displayed a lower CD86/CD163 ratio and expressed more CD163 receptors. In addition, the cells showed reduced phagocytic capacity, compared to Ctr cells, suggesting that LUSO might modulate macrophage phenotype towards M2, without additional M2 inducers. Moreover, our results strongly suggested that LUSO NMW shifted LPS‐induced M1 macrophages into M2, as supported by the reduction in the pro‐inflammatory parameters analyzed (i.e., *Il6* gene expression; IL‐1β, IL‐6 and TNF‐α secretion levels), and the increase in the antioxidant enzyme HMOX1, at the transcriptional and post‐transcriptional level (Created with Microsoft Office PowerPoint).

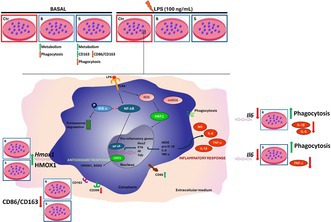

## 1 Introduction

Acute inflammation is an important and complex process carried out by the organism to restore homeostasis. It occurs in two phases: (1) the initiation phase, when the innate immune system is activated by tissue damage (sterile inflammation) or pathogens; and (2) the resolution phase, when early and highly regulated cellular and biochemical processes are triggered to counteract inflammation. These include macrophages shifting from a pro‐inflammatory (M1) to an anti‐inflammatory (M2) phenotype, clearance of the injured site through phagocytosis, reduction of pro‐inflammatory cytokines, and tissue regeneration (Alfaro et al. 2022).

Macrophages are a highly heterogeneous and multifunctional population of immune cells that participate in all phases of the immune response. These cells are the first to respond when pattern recognition receptors (PRR) (e.g., Toll‐like receptor (TLR) 4 activation by LPS) or cytokine receptors are stimulated, triggering an inflammation cascade of events that involves nuclear translocation and activation of Nuclear factor kappa B (NF‐κB), inducing the transcription of primary inflammatory response genes such as *Tnfa* and *Il1b* among several others (e.g., *Nos2*, *Il6*, Prostaglandin‐endoperoxide synthase 2, *Ptgs*2) (Liu et al. 2017; Dorrington and Fraser 2019).

In a simplistic way, macrophages can adopt a pro‐inflammatory (M1) or anti‐inflammatory (M2) phenotype depending on being activated through the classical or alternative (also known as reparative) pathway, respectively (de Sousa et al. 2019). M1 macrophages polarization is induced by pro‐inflammatory stimuli like the cytokines interferon‐γ (IFN‐γ) and tumor necrosis factor‐alpha (TNF‐α), or bacterial LPS. These effector cells release high levels of pro‐inflammatory cytokines and express high levels of inducible nitric oxide synthase (iNOS), and nitric oxide (NO), since NO as well as reactive oxygen species (ROS), are important mediators for the microbicidal activity of M1 macrophages. The expression of CD86 costimulatory molecule is often associated with an M1 phenotype (de Sousa et al. 2019). In contrast, M2 macrophages are involved in the resolution phase of inflammation and immune response suppression, producing high levels of anti‐inflammatory cytokines (Strizova et al. 2023). The expression of CD206 (mannose receptor) and CD163 (haptoglobin‐hemoglobin complexes scavenger receptor) is usually correlated to the M2 phenotype (de Sousa et al. 2019; Strizova et al. 2023). However, it has been noticed that these cells respond to microenvironmental stimuli with a high level of plasticity, exhibiting several different immunophenotypes and functional properties, that sometimes overlap (M1 and M2 polarization with M2 macrophages subsets depending on their cell surface molecules, genes, and specific functions are detailed elsewhere) (de Sousa et al. 2019; Strizova et al. 2023).

Acute inflammation is usually temporary but may turn chronic when the resolution phase fails and has been associated with the development of several different pathologies (Alfaro et al. 2022). In the past decades, increasing reports conducted in cells (Trapecar et al. 2012; Aversano et al. 2020; Barnich et al. 2021), animal models (Pereira et al. 2015; Lalovic et al. 2020; Budinskaya et al. 2022), and humans (Rylander and Arnaud 2004; Kitagawa et al. 2011; Dupont et al. 2014; Jovicic et al. 2023) have demonstrated the positive role of NMW ingestion in health, mostly associated with an anti‐inflammatory effect, particularly when containing carbonic metabolites and/or sulphates and/or iron, which are not observed in tap water (Quattrini et al. 2016). ‘Natural mineral water’ is defined by the European legislation (2009/54/EC Directive) as a “microbiologically wholesome water (…) originating in an underground water table or deposit and emerging from a spring tapped at one or more natural or bore exits”. NMW can be distinguished from tap water by its original purity and nature (mineral content, trace elements, or other constituents, and eventually, health effects), which are preserved intact because of its protected underground origin. NMW can contribute to important minerals intake that are better absorbed than those in foods, which might explain its extensive use in human nutrition with widely recognized health‐related properties (Shenkin 2003; Stefanache et al. 2023).

In Portugal, there are ~50 thermal centers, with a high diversity of NMW due to geological variability (Rebelo et al. 2015; Araujo et al. 2017). These waters exhibit different therapeutic properties that have long been empirically recognized. However, the scientific validation supporting this empirical knowledge is still scarce. In a pioneer study, we previously showed that 11 out of 14 Portuguese NMW displayed anti‐inflammatory properties in vitro (Silva et al. 2020), where it was shown that LUSO NMW (formerly denoted as Chlorinated/Sodic, CS) has reduced LPS‐induced NO accumulation and iNOS expression and increased the NO scavenging activity of macrophages. Therefore, in the present work, we aimed to study the effect of LUSO NMW on macrophage cells' functional activity, which might underlie its attributable anti‐inflammatory properties. According to the 2023 official water chemical analysis (Table S1), LUSO NMW has an acidic pH (~5.15), low mineralization (thus considered hyposaline), with a concentration of silica of about 26% of total mineralization, and is mainly composed of chlorine and sodium. As stated by the Portuguese Ministry of Health, LUSO NMW‐based therapies are primarily indicated for the Respiratory System, Rheumatic and Musculoskeletal disorders, the Circulatory System, and the Nephro‐Urinary System (which are often associated with an inflammatory status), with water intake being the fundamental key in LUSO NMW‐based therapies. Thus, we studied the effect of LUSO NMW (collected directly from Spring or bottled) in RAW 264.7 macrophages exposed to LPS (an outer membrane component of the Gram‐negative bacteria cell wall that induces inflammation through TLR4). Macrophages' metabolism, M1 (pro‐inflammatory) and M2 (anti‐inflammatory) profile, inflammatory and antioxidant gene expression and protein levels, NO and inflammatory cytokines secretion levels, and phagocytic capacity were investigated.

## 2 Material and Methods

All reagents were from Sigma‐Aldrich unless otherwise stated.

### 2.1 LUSO NMW Collection

Bottled LUSO NMW (commercially available for human consumption) and Spring LUSO NMW were used for comparison. Spring NMW was collected from the spring after purging, according to proper procedures, as described in Silva et al. (2020). Briefly, NMW was collected in sterile flasks (VWR, #331‐0278) that were maintained at 4°C, in the dark, until use. Each flask was opened once to avoid NMW composition alterations. One of the flasks was used to measure NMW pH and organoleptic features (i.e., odor, color, deposit and aspect). All other physicochemical analyses (Table S1) were periodically measured at the thermal center. The physicochemical analyses of Bottled NMW, collected and bottled as described by Vega (2022), were performed before bottling.

### 2.2 Cell Culture and Treatment

Raw 264.7 (ATCC, #TIB‐71) mouse macrophage cell line, was cultured in DMEM (powder, low glucose with pyruvate and L‐Glutamine; Gibco, #31600083. Detailed composition available at https://www.thermofisher.com/pt/en/home/technical‐resources/media‐formulation.51.html), supplemented with 10% (v/v) of inactivated FBS (Biowest, #S1810‐500), 100 U/mL penicillin and 100 μg/mL streptomycin (Grisp, #GTC05.0100), D‐Glucose (up to the final concentration of 4.5 g/L) and 1.5 g/L sodium bicarbonate, at 37°C in a humidified atmosphere of 95% air and 5% CO_2_. Before reaching confluence (every 2 days), the cells were passaged using a cell scrapper to detach the cells, which were further cultured in fresh culture media according to ATCC recommendations. The cells were tested for mycoplasma contamination and were used between passage numbers 20–30 (reported to be the most stable timeframe for these cells) (Taciak et al. 2018).

For the experiments, the cells were exposed to culture medium prepared using (1) ultrapure water (control, Ctr); (2) NMW collected directly from Spring (S) or (3) Bottled NMW (B), previously filtered with a sterile 0.2 μm membrane, and with adjusted pH to 7.2, to mimic the physiologic pH. After 24 h, the medium was removed and replaced with fresh medium, for another 24 h, in the absence or presence of LPS from *Escherichia coli* (serotype 026:B6; Sigma‐Aldrich, #L2654). 100 ng/mL LPS were added to the cells for the time referred to in each method and depicted in the figures. In all experiments, the cells were maintained in NMW‐containing medium for 48 h.

### 2.3 Cell Metabolism (Resazurin Assay)

Cell metabolism was assessed using resazurin reduction colorimetric assay, as previously described (O'Brien et al. 2000). Briefly, 50,000 cells were plated in a 96‐well plate, with a final volume of 0.2 mL/well, in duplicates, for 48 h, without or with LPS (100 ng/mL; 24 h). The medium was further replaced with medium containing 50 μM of resazurin solution (in sterile PBS). After 1 h of incubation, absorbance was read at 570 and 620 nm with a Synergy HT multimode microplate reader (BioTek). Results were expressed as percentage (%) of control (Ctr).

### 2.4 Macrophages Polarization

Pro‐inflammatory and anti‐inflammatory macrophage polarization was inferred by staining the cells for M1‐ and M2‐associated surface markers. Briefly, the cells (1 × 10^6^) were plated in a 6‐well plate, with a final volume of 2 mL/well, for 48 h, without or with LPS (100 ng/mL; 24 h). Next, the cells were collected, centrifuged (300 × g for 5 min, at 4°C), and washed (twice) with PBS (pH 7.4). The pellet was further resuspended in 400 μL of PBS/FBS (1%), and 100 μL of cell suspension was incubated with 3 μL (0.6 μg) of the antibodies against the M2 markers PerCP/Cyanine5.5 anti‐mouse CD206 (MMR) (Biolegend, #141715) and APC anti‐mouse CD163 (Biolegend, #155305), and the M1 marker PE anti‐mouse CD86 (Biolegend, #159203), for 30 min, at 4°C, in the dark. 100 μL of cell suspension (from each experimental condition) were maintained in PBS and left unstained to be used as a fluorescence‐negative control. At the end of the incubation, 500 μL of PBS was added to the tubes, and the cells were centrifuged (300 × g for 5 min, at 4°C). The supernatant was discarded, and the pellet resuspended in 100 μL of PBS/FBS (1%). The cells were analyzed by Flow Cytometry, in a CytoFLEX cytometer (Beckman Coulter). Forward and side‐scatter gate was set to exclude dead cells. Additionally, a gate along the diagonal in a SSC‐A/SSC‐H dot plot was used to select the single cells (representative dot plots of unstained cells used to gate positive staining are shown in Figure S1A,B). The results are shown as the % of positive cells for each marker and the Mean Fluorescence Intensity (MFI) associated with the cell population (stained cells—unstained cells). The pro‐inflammatory/anti‐inflammatory MFI ratios (CD86/CD206 and CD86/CD163) are also presented as a more accurate indicator of cell phenotype (Liu et al. 2020).

### 2.5 NF‐kB Nuclear Translocation

To evaluate the presence of NF‐κB in the nucleus, the cells (25,000) were plated in a 1 μ‐Slide 8 Well ibiTreat (Ibidi, #80806), with a final volume of 300 μL/well, for 48 h, without or with LPS (100 ng/mL; 1 h). Then, cells were washed with ice‐cold PBS (pH 7.4) and fixed with 4% paraformaldehyde (PFA) for 15 min at room temperature (RT). After a washing step (three times, 5 min each) with 0.1 M glycine (in PBS), the cells were incubated with a blocking solution (10% BSA +0.3% Triton, in PBS) for 1 h at RT. The blocking solution was removed, and the cells were incubated with a rabbit monoclonal anti‐NF‐κB p65 (D14E12) XP antibody (Cell Signaling Technology, #8242S; 1:400) in PBS/BSA (1%) overnight (ON) at 4°C. After a washing step with PBS, the cells were further incubated with anti‐rabbit IgG (H + L) CF488A (Sigma‐Aldrich, #SAB4600045; 1:400) in PBS/BSA (1%) and Alexa Fluor555 Phalloidin (Invitrogen, Thermo Fisher Scientific, #A34055; 1:1000) in PBS/BSA (1%) for 1 h at RT. The cells were further washed, and the nuclei stained with 0.5 ng/mL Hoechst 33342 (Invitrogen, ThermoFisher Scientific, #H3570). After a washing step with PBS, preparations were mounted with Ibidi Mounting Medium (Ibidi, #50001), and the cells were visualized by fluorescence microscopy in a confocal microscope Zeiss LSM 710 (Carl Zeiss AG) with a Plan‐Apochromat 40×/1.4 Oil DIC M27 objective. Specificity was evaluated in negative controls by omitting the first antibody (Figure S2). Images were analyzed using ImageJ (version 1.53c) software, and the results are expressed as Mean Fluorescence Intensity (MFI).

### 2.6 Gene Expression

The cells (1 × 10^6^) were plated in a 6‐well plate, with a final volume of 2 mL/well, for 48 h, without or with LPS (100 ng/mL; 6 or 18 h). Next, the cells were washed with ice‐cold PBS, and total RNA was extracted with NZYol (NZYtech, #MB18501) reagent. RNA concentration was quantified using a NanoDrop spectrophotometer (Thermo Scientific). Samples were stored at −80°C in an RNA storage solution until use. 2 μg of RNA were transcribed to cDNA using the NZY First‐Strand cDNA Synthesis kit (NZYtech, #MB12501), according to the manufacturer's protocol, in a C1000 Thermal Cycler (Bio‐Rad). The resulting products were amplified in duplicate with NZYSpeedy qPCR Green Master Mix (2×) kit (NZYtech, #MB22403) by real‐time reverse transcription‐quantitative polymerase chain reaction (RT‐qPCR) in a CFX Connect Real‐Time System (Bio‐Rad).

The thermocycling protocol was as follows: 95°C for 2 min (1 cycle); 95°C for 5 s, 60°C for 10 s, 72°C for 20 s (cycling process repeated 40 times); 55°C–95°C (with 0.5°C increment; 1 cycle). To standardize the results, the 2^−ΔΔCq^ method was applied (Livak and Schmittgen 2001) with *Hprt1* (Hypoxanthine‐guanine phosphoribosyl transferase) as the reference gene (which was stably expressed in all experimental conditions; Figure S3). The results were analyzed using Bio‐Rad CFX Maestro 2.3 system software (Bio‐Rad). Mouse forward and reverse primers (Sigma‐Aldrich) are listed in Table S2.

### 2.7 Cell Lysates and Western Blotting

To determine protein expression, total cell lysates, protein quantification and Western blotting (WB) were performed as previously described by us (Silva et al. 2020). 1 × 10^6^ cells were plated in a 6‐well plate, with a final volume of 2 mL/well, for 48 h, without or with LPS (100 ng/mL; 24 h). After, the cells were washed three times with ice‐cold PBS, detached with a cell scraper, and centrifuged (500 × g, 5 min, at 4°C). The pellet was incubated with RIPA buffer (150 mM NaCl; 50 mM Tris–HCl, pH 8.0; 1% Nonidet P‐40; 0.5% sodium deoxycholate (v/v); 0.1% sodium dodecyl sulfate (SDS) (v/v); 2 mM EDTA), supplemented with 1 mM DTT, protease (cOmplete Mini, Roche, #04693124001) and phosphatase (PhosSTOP, Roche, #04906837001) inhibitor cocktails (as recommended by the manufacturer). After 30 min on ice, the cells were centrifuged (12,000 × g for 10 min, at 4°C), and protein concentration was calculated through the bicinchoninic acid method (BCA). Lysate samples were denatured in sample buffer (0.125 mM Tris, pH 6.8; 2% SDS (w/v); 100 mM DTT; 10% glycerol (v/v) and bromophenol blue), at 95°C, for 5 min. 20 μg of protein were separated on a 10% SDS‐polyacrylamide gel and transferred to a PVDF membrane (Millipore). Membranes were blocked with 5% fat‐free dry milk (w/v) in TBS‐T (Tris‐buffered saline with 0.1% Tween 20 (v/v)), for 1 h, at RT and incubated with the primary antibodies: mouse monoclonal anti‐iNOS (R&D Systems, #MAB9502; 1:500), rabbit polyclonal anti‐IL‐1 beta (Abcam, #ab9722; 1:1000) and mouse monoclonal anti‐HMOX1 (Invitrogen, Thermo Fisher Scientific, #MA1‐112; 1:500), ON at 4°C. After washing with TBS‐T, the membranes were incubated with the horseradish peroxidase‐conjugated secondary antibodies anti‐mouse IgG, HRP‐linked Antibody (Cell Signaling Technology, #7076; 1:2000) or anti‐rabbit IgG, HRP‐linked Antibody (Cell Signaling Technology, #7074; 1:2000), for 1 h at RT. Mouse monoclonal anti‐β‐Tubulin (Sigma‐Aldrich Chemical, #T7816; 1:20,000) was used as a loading control. Blots were visualized by chemiluminescence with Clarity Western Enhanced Chemiluminescent Substrate agent (Bio‐Rad, #1705061) in ImageQuant LAS 500 (GE Healthcare) apparatus and analyzed using TotalLab TL120 software.

### 2.8 Determination of Nitric Oxide (NO) Production and Cytokines Secretion

NO and cytokines levels were assessed in the supernatant of the cultures used to determine protein levels by WB. NO production was inferred by measuring nitrite accumulation through the Griess colorimetric assay (Green et al. 1982). Briefly, equal volumes of cell culture supernatants and Griess reagent [1% sulphanilamide (w/v) in 5% phosphoric acid (w/v) and 0.1% N‐(1‐naphthyl)‐ethylenediamine dihydrochloride (w/v)] were mixed and incubated at RT for 30 min. The absorbance was measured at 550 nm in a Synergy HT multimode microplate reader (BioTek). Nitrite concentration was calculated through regression analysis of a sodium nitrite standard curve. Results are expressed as % of NO produced in LPS‐treated cells.

IL‐1β, IL‐6 and TNF‐α supernatant levels were determined using the IL‐1 beta mouse ELISA Kit (Invitrogen, Thermo Fisher Scientific, #BMS6002‐2), mouse IL‐6 ELISA Kit (Invitrogen, Thermo Fisher Scientific, #KMC0061) and the mouse TNFα High Sensitivity ELISA Kit (Invitrogen, Thermo Fisher Scientific # BMS607‐2HS), according to the manufacturer's instructions. The results were expressed in pg/ml.

### 2.9 Phagocytosis Assay

To determine the phagocytic capacity of the macrophages, 50,000 cells were plated in a 6‐well plate, with a final volume of 2 mL/well, for 48 h, without or with LPS (100 ng/mL; 24 h). After, latex beads (amine‐modified polystyrene, fluorescent yellow‐green; size 1 μm; Sigma, #L1030) were delivered to the cells at 1:10 ratio (cells: beads), for 2 h, at 37°C (in one Ctr the beads were washed after 3 min—*Time 0*, as fluorescence‐negative control). As a negative control for beads internalization, Ctr cells were treated as previously mentioned but kept on ice during beads incubation (*Ice*) (Lam et al. 2016). At the end of the incubation period, the cells were washed three times with PBS (pH 7.4) and analyzed by Flow Cytometry, in a CytoFLEX cytometer (Beckman Coulter). Forward and side‐scatter gate was set to exclude dead cells. Additionally, a gate along the diagonal in a SSC‐A/SSC‐H dot plot was used to select the single cells (representative dot plots of cell populations not exposed to fluorescent beads and used to gate positive internalization are shown in Figure S4). The results are shown as the % of positive cells that internalized beads (phagocytosis affinity) and the Mean Fluorescence Intensity (MFI) associated with the cell population (cells exposed to beads—cells not exposed to beads), representing phagocytosis capacity (Suleimanov et al. 2024). For each experimental condition (Ctr, B and S NMW cell cultures), the LPS‐treated/untreated cells ratio was calculated to evaluate the fold increase of LPS‐induced phagocytosis.

### 2.10 Statistical Analysis

The results are presented as mean ± standard error of the mean (SEM) of the indicated number of experiments and were analyzed with one‐way ANOVA with Dunnett or Tukey multiple comparison test to evaluate differences between three or more groups. Student's *t*‐test were also used to disclose significant differences between two specific groups. The software used was GraphPad Prism (version 8.0.2; www.graphpad.com). *p* < 0.05 was considered significant.

## 3 Results

### 3.1 LUSO NMW Increased RAW 264.7 Macrophages' Metabolic Capacity

**FIGURE 1:**
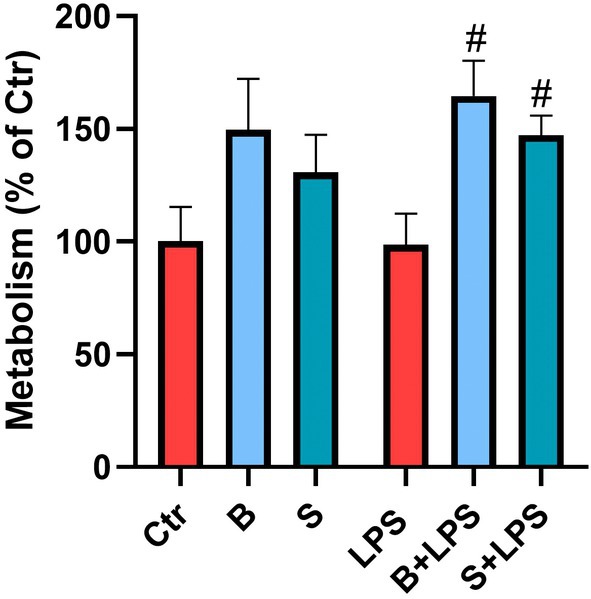
LUSO NMW increased RAW 264.7 cells' metabolism. Macrophage metabolism was assessed through the resazurin assay. Data corresponds to the mean ± SEM of four independent experiments and are represented as % of control (Ctr). Statistics: One‐way ANOVA with Dunnett's multiple comparisons test. *p* < 0.05 was considered significant. # *p* < 0.05, compared to LPS.

The effect of LUSO NMW (pH 7.2) on macrophage metabolism was evaluated using the resazurin assay, which assesses general metabolic activity and is commonly used as an indirect indicator of cell viability. The cells were cultured in normal culture medium (control, Ctr), bottled (B)‐ or Spring (S)‐containing medium (for 48 h), in the absence or presence of the pro‐inflammatory stimulus LPS (added for 24 h). Our results showed that LUSO NMW significantly stimulated cell metabolism in inflammatory experimental conditions (Figure 1; B + LPS and S + LPS compared to LPS).

Although not statistically significant, a clear qualitative increase in the metabolic capacity of cells cultured with B and S NMW (1.5 and 1.3 fold‐increase, respectively, compared to Ctr) is also observed, suggesting that both B and S LUSO NMW boost macrophages' metabolic state, independently of the cells' milieu.

### 3.2 LUSO NMW Promoted RAW 264.7 Macrophage Polarization Towards an Anti‐Inflammatory (M2) Phenotype

**FIGURE 2:**
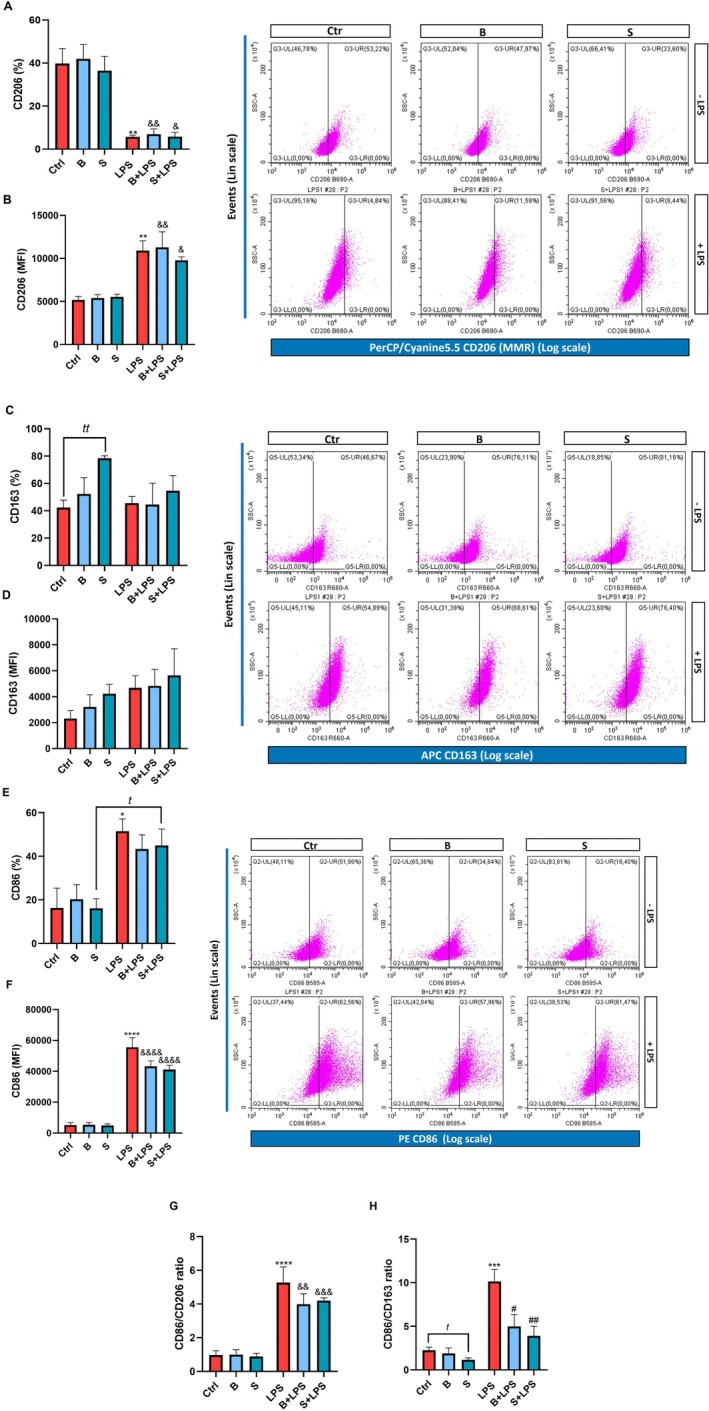
LUSO NMW promoted RAW 264.7 macrophage anti‐inflammatory phenotype. (A, B) Expression of the M2 marker CD206, (C, D) expression of the M2 marker CD163, (E, F) expression of the M1 marker CD86 determined by flow cytometry. Representative dot plots of the experiment are presented. Data corresponds to the mean ± SEM of four independent experiments and are represented as % of cells (A, C, E) and MFI (B, D, F). (G) CD86/206 and (H) CD86/163 MFI ratios. Statistics: One‐way ANOVA with Tukey's multiple comparisons test and unpaired *t*‐test (*t*). *p* < 0.05 was considered significant. * *p* < 0.05, ** *p* < 0.01, *** *p* < 0.001 and **** *p* < 0.0001, compared to Ctr; & *p* < 0.05, && *p* < 0.01, &&& *p* < 0.001 and &&&& *p* < 0.0001 compared to the control cells of B and S (without LPS); # *p* < 0.05 and ## *p* < 0.01, compared to LPS; *t p* < 0.05 and *tt p* < 0.01. Lin – Linear and Log – Logarithmic (dot plots YY axis).

The effect of B and S LUSO NMW on macrophage profiles M1 (pro‐inflammatory) or M2 (anti‐inflammatory) was investigated. Hence, the cells were cultured in the different culture media alone (for 48 h) or with LPS (a classical M1 inducer) for 24 h. Expression of the surface markers CD206 and CD163 (M2), and CD86 (M1), was determined by flow cytometry, and the results are presented as % of positively stained cells and mean intensity fluorescence (MFI), which indicates how strongly the marker is expressed by the cells (Figure 2).

At basal conditions, LUSO NMW did not affect macrophages CD206 (Figure 2A,B) and CD86 (Figure 2E,F) expression. In contrast, S NMW significantly increased the % of cells expressing CD163 compared to Ctr (Figure 2C), with a non‐significant 1.8‐fold increase in the MFI (Figure 2D). Moreover, LPS induced a reduction in the % of CD206 positive cells (Figure 2A), although with high intensity (Figure 2B). As expected, LPS increased both the % of CD86‐positive cells and the MFI in Ctr, S, and B NMW cell cultures (Figure 2E,F), although B + LPS lacks statistical significance (compared to B; Figure 2F). Of notice, B + LPS and S + LPS displayed lower MFI levels than LPS‐exposed cells (1.2‐fold and 1.3‐fold decrease, respectively, compared to LPS; Figure 2F), suggesting that LPS induced macrophages to acquire an M1 phenotype that was partially mitigated by B and S NMW.

Due to the heterogeneity of macrophage populations, with classical M1 and M2 markers being expressed in different phenotypes (Strizova et al. 2023), we calculated the ratio between pro‐ and anti‐inflammatory markers, which is a more accurate indication of macrophage profile. As expected, LPS increased both CD86/CD206 (Figure 2G) and CD86/CD163 (Figure 2H) ratios, reflecting the M1 status of the cells, regardless of the culture medium they were exposed to. However, the CD86/CD206 ratio tended to decrease in cells cultured in B + LPS and S + LPS NMW (1.3‐fold and 1.2‐fold decrease, respectively, compared to LPS; Figure 2G), and the CD86/CD163 ratio was significantly lower in these cell cultures (B + LPS and S + LPS compared to LPS; Figure 2H). Interestingly, CD86/CD163 was also significantly decreased in S NMW cell cultures without LPS (compared to Ctr; Figure 2H). Altogether, these results indicate that LUSO NMW reduces LPS‐induced M1 phenotype, and that S NMW promotes an M2 phenotype in macrophages under homeostatic conditions.

### 3.3 LUSO NMW Did Not Affect LPS‐Induced NF‐κB Nuclear Translocation but Decreased Inflammation Mediators in RAW 264.7 Macrophages

**FIGURE 3:**
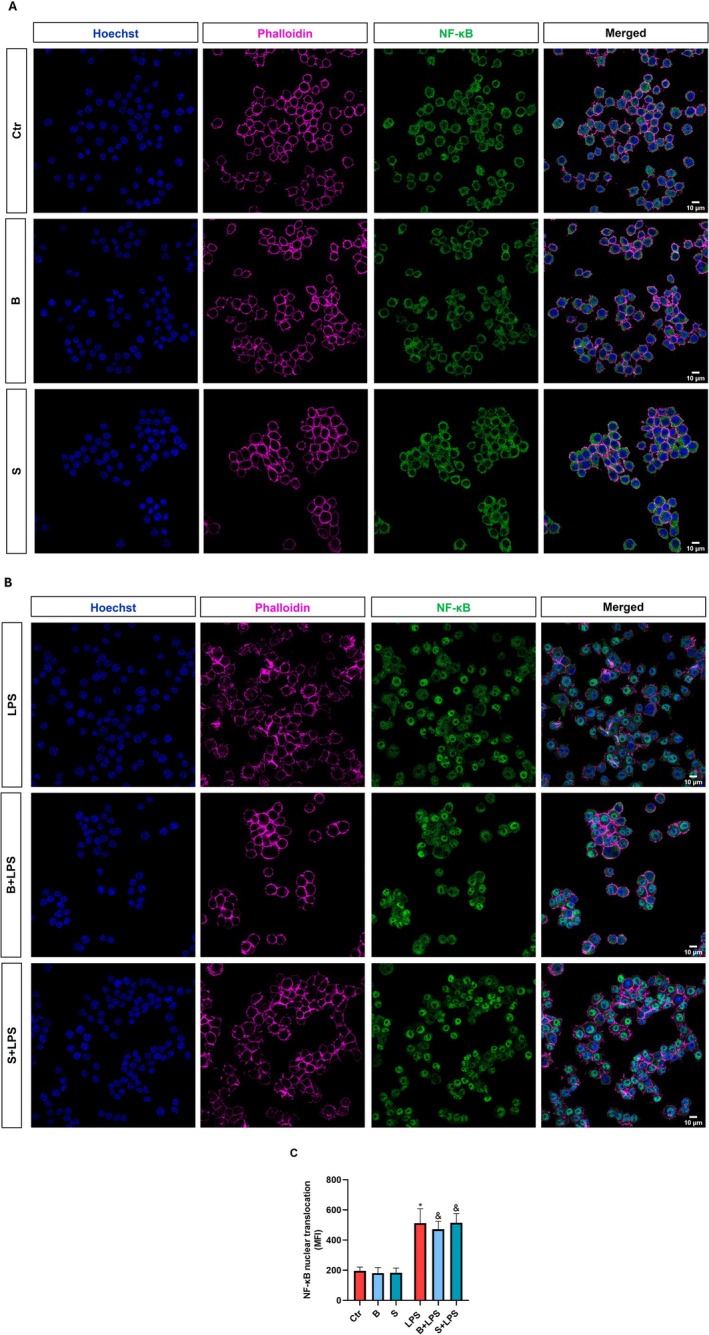
LPS‐induced NF‐κB nuclear translocation was not affected by LUSO NMW in RAW 264.7 macrophages. (A–C) Immunofluorescence images and analysis of NF‐κB (green) nuclear translocation, with F‐Actin (phalloidin, magenta) and nuclear staining (Hoechst, blue), evaluated by confocal microscopy. Representative images of the experiments without LPS (A) and with LPS (B) are presented. Scale bar—10 μm; Magnification 40×. Data correspond to the mean ± SEM of three independent experiments and are represented as MFI (C). Statistics: One‐way ANOVA with Tukey's multiple comparisons test. *p* < 0.05 was considered significant. * *p* < 0.05, compared to Ctr; & *p* < 0.05, compared to the control cells of B and S (without LPS). Total cell number analyzed: Ctr = 944 cells; B = 985 cells; S = 892 cells; LPS = 1223 cells; B + LPS = 1004 cells; S + LPS = 1105 cells.

Inflammation is tightly related to the NF‐κB signaling pathway. Therefore, macrophages were exposed to the different culture media (for 48 h), with or without LPS (for 1 h, chosen after assessing LPS‐induced Ik‐Bα phosphorylation levels by WB; Figure S5). After, the cells were stained against NF‐κB (green), phalloidin (magenta), an F‐actin filament marker, and Hoechst (blue), for nucleus visualization. Cells' immunofluorescence images were obtained by confocal microscopy, and the MFI was analyzed (Figure 3).

As shown in Figure 3, LPS similarly induced NF‐κB nuclear translocation, either in cells cultured in normal culture medium (LPS) or in medium containing Bottled (B + LPS) or Spring (S + LPS) LUSO NMW (Figure 3C).

**FIGURE 4:**
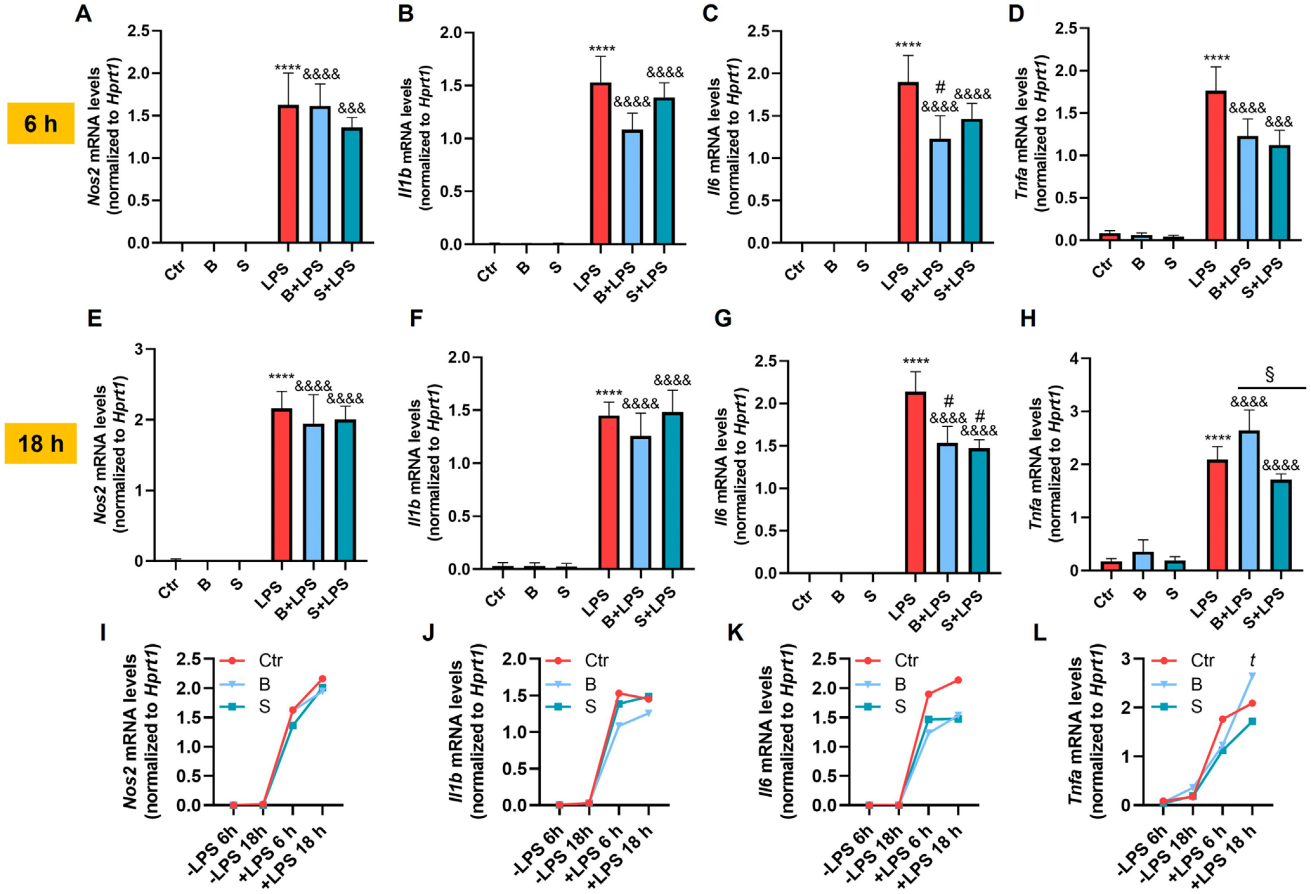
LUSO NMW down‐regulated *Il6* in RAW 264.7 macrophages. (A–H) RT‐qPCR analysis of the pro‐inflammatory genes *Nos2*, *Il1b*, *Il6*, and *Tnfa* after 6 h (A–D) and 18 h (E–H) of LPS incubation. (I–L) The graphs depict a comparison between gene expression at 6 and 18 h. Data correspond to the mean ± SEM of six independent experiments and are normalized to the reference gene *Hprt1*. Statistics: One‐way ANOVA with Tukey's multiple comparisons test and unpaired *t*‐test (*t*). *p* < 0.05 was considered significant. **** *p* < 0.0001, compared to Ctr; &&& *p* < 0.001 and &&&& *p* < 0.0001, compared to the control cells of B and S (without LPS); # *p* < 0.05, compared to LPS; *t p* < 0.05, compared to 6 h; § *p* < 0.05.

We further analyzed NF‐κB‐dependent gene expression in cells cultured in regular medium (Ctr) or LUSO NMW‐media (B and S), alone (for 48 h) or with LPS. We chose 6 and 18 h of LPS stimulation, as representative time points of the peak of inflammatory genes response (6 h), and the beginning of the “resolution” phase (18 h), before the feedback control is completed (24 h) (Schroder et al. 2012). The expression of the pro‐inflammatory genes *Nos2* (which encodes iNOS; Figure 4A,E), *Il1b* (which encodes IL‐1β; Figure 4B,F), *Il6* (which encodes IL‐6; Figure 4C,G), and *Tnfa* (which encodes TNF‐α; Figure 4D,H) was determined by RT‐qPCR.

According to Figure 4, the expression of the genes analyzed was barely detected without the inflammatory stimulus and was induced after 6 h (Figure 4A–D) and 18 h (Figure 4E–H) of LPS exposure, in all experimental conditions. Except for *Il6* mRNA levels, which decreased in cells exposed to B + LPS at 6 h (Figure 4C) and in cells exposed to B + LPS and S + LPS at 18 h (Figure 4G), compared to LPS, no other significant differences were observed. Yet, a trend towards reduction of *Tnfa* mRNA levels in cells cultured with B and S NMW was observed after 6 h of LPS exposure (1.4‐fold and 1.5‐fold, respectively), compared to LPS (Figure 4D). However, after 18 h, B + LPS‐exposed cells displayed increased *Tnfa* mRNA levels, compared to S + LPS (Figure 4H), reflecting the augmentation in *Tnfa* gene expression over time, in macrophages cultured with B + LPS (+LPS 6 h vs. + LPS 18 h; Figure 4L).

**FIGURE 5:**
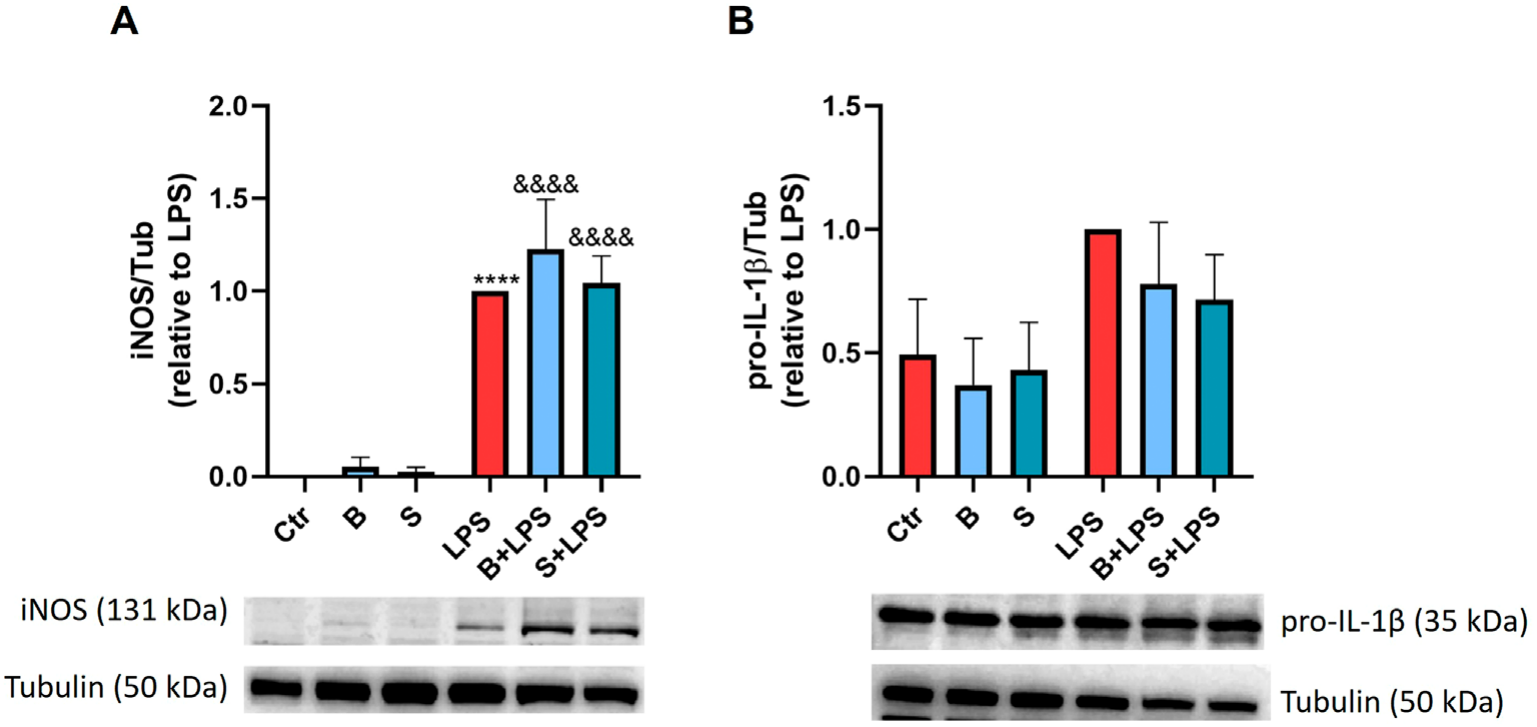
LUSO NMW did not affect pro‐inflammatory protein levels in RAW 264.7 macrophages. (A) iNOS, and (B) pro‐IL‐1β protein levels determined by WB. Representative blot images are presented (original blots are shown in Figure S7). Data corresponds to the mean ± SEM of six independent experiments and are expressed relatively to LPS. Statistics: One‐way ANOVA with Tukey's multiple comparisons test. *p* < 0.05 was considered significant. **** *p* < 0.0001, compared to Ctr; &&&& *p* < 0.0001, compared to the control cells of B and S (without LPS).

We also analyzed the protein levels of iNOS (classically induced by LPS) and pro‐IL‐1β, both proteins produced by activated macrophages and important mediators of the inflammatory response (Liu et al. 2017; Dorrington and Fraser 2019). Hence, macrophages were exposed to the different culture media (for 48 h), with or without LPS (for 24 h), and the protein levels were determined by WB (Figure 5).

**FIGURE 6:**
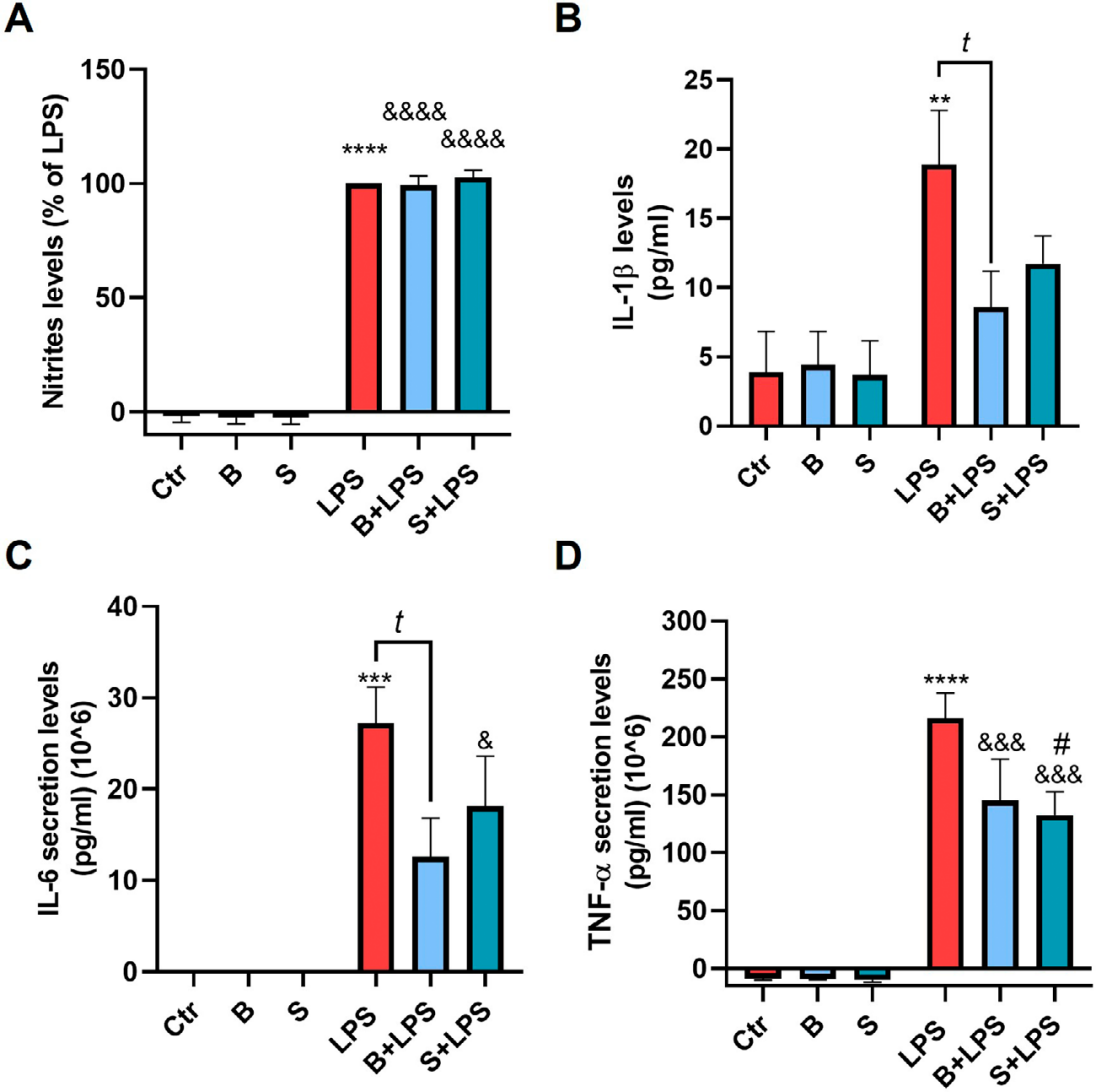
LUSO NMW decreased the secretion of inflammatory proteins in RAW 264.7 macrophages. (A) Nitrites levels (determined by Griess assay), (B) IL‐1β, (C) IL‐6, and (D) TNF‐α supernatant levels determined by commercial kits. Data correspond to the mean ± SEM of eight independent experiments (A, expressed as % of LPS) and four independent experiments (B–D, expressed in pg/ml). Statistics: One‐way ANOVA with Tukey's multiple comparisons test and unpaired *t*‐test (*t*). *p* < 0.05 was considered significant. ** *p* < 0.01, *** *p* < 0.001 and **** *p* < 0.0001, compared to Ctr; & *p* < 0.05, &&& *p* < 0.001, and &&&& *p* < 0.0001, compared to the control cells of B and S (without LPS); # *p* < 0.05, compared to LPS; *t p* < 0.05.

According to the results, neither B nor S NMW‐medium decreased iNOS levels induced by LPS (Figure 5A). Similarly, no differences were detected in pro‐IL‐1β protein levels, which were slightly and equally augmented by LPS. These results align with those of gene expression (Figure 4A,B,E,F, respectively), suggesting that LUSO NMW does not affect the intracellular levels of these two inflammatory proteins. We further quantified nitrites levels (through Griess assay, to infer about NO production), and the cytokines IL‐1β, IL‐6, and TNF‐α (by ELISA), in the cell cultures supernatant (Figure 6). It is important to notice that according to published research RAW 264.7 cells do not produce or secrete IL‐1β (Hazuda et al. 1990; Pelegrin et al. 2008) due to the lack of ASC (Pelegrin et al. 2008), an important component of the NLRP3 inflammasome responsible for the production of the 17 kDa mature protein. However, since RAW macrophages can secrete pro‐IL‐1β, which can be processed extracellularly by proteases into more potent forms (Hazuda et al. 1990; Pelegrin et al. 2008), we proceeded with the quantification of IL‐1β in the supernatant.

As depicted in Figure 6A, LPS induced a similar increase in NO production in all experimental conditions, corroborating the observed *Nos2* gene expression (Figure 4A–E) and iNOS protein levels (Figure 5A). As expected, the levels of the cytokines analyzed were increased in the supernatant of LPS‐exposed control cells (LPS compared to Ctr; Figure 6B–D). IL‐6 and TNF‐α secreted levels were also significantly augmented by LPS in LUSO NMW cell populations (S vs. S + LPS, Figure 6C; B vs. B + LPS and S vs. S + LPS, Figure 6D, respectively), though to a lower extent. In fact, in the B + LPS‐exposed cells supernatant, we observed significantly lower levels of IL‐1β and IL‐6 (compared to LPS; Figure 6B,C, respectively), and S + LPS cells secreted significantly lower levels of TNF‐α (compared to LPS; Figure 6D). Altogether, these results demonstrate that LUSO NMW decreases the gene and mature protein levels of relevant pro‐inflammatory mediators, suggesting its anti‐inflammatory effect.

In the previous experiments where the effect of NMW on LPS‐induced pro‐inflammatory proteins was investigated (Figures 5 and 6), we also used Indomethacin (Sigma, #I7378), a nonsteroidal anti‐inflammatory drug, as a control (Indo 10 μg/mL; Figure S6). Indo decreased iNOS expression but did not affect IL‐1β protein levels (Figure S6A,B, respectively). In addition, Indo reduced IL‐6 and increased IL‐1β mature protein levels (Indo+LPS vs. LPS; Figure S6C,D, respectively), as observed by Liu et al. (2022) in RAW macrophages exposed to 1 μg/mL of LPS and 10 μg/mL of Indo, for 24 h. However, while the authors reported a reduction in TNF‐α levels, no differences were observed in our study (Indo+LPS vs. LPS; Figure S6E). Although we cannot compare the degree of effect of an NMW with a pharmacological drug, these results support and validate the experiments performed and the results obtained.

### 3.4 LUSO NMW Promoted the Expression of the Antioxidant Enzyme HMOX1 in RAW 264.7 Macrophages

**FIGURE 7:**
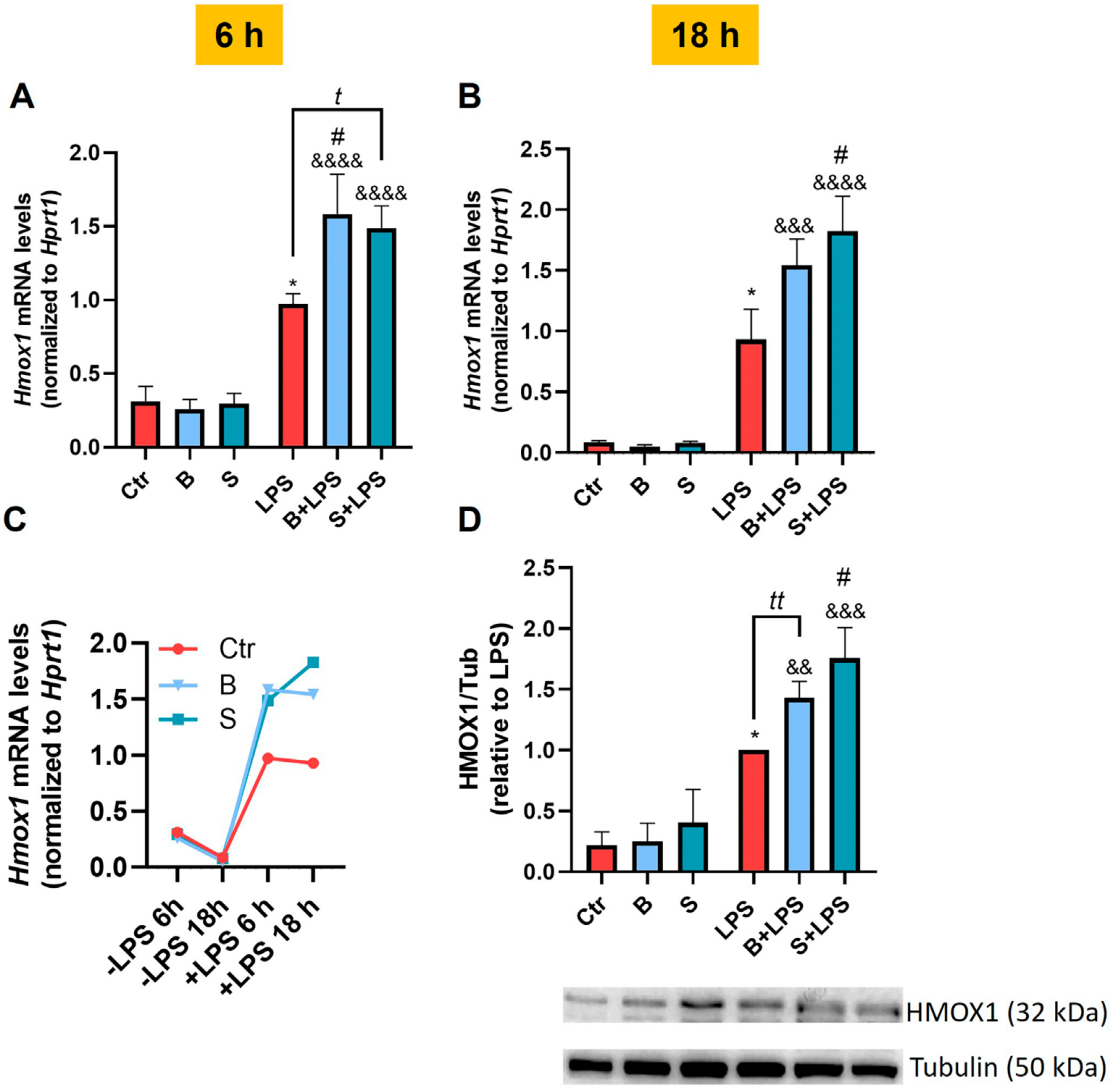
LUSO NMW increased gene and protein levels of HMOX1 in RAW 264.7 macrophages. (A, B) Gene expression of the antioxidant enzyme HMOX1 after 6 h (A) and 18 h (B) of LPS incubation was determined by RT‐qPCR. (C) The graph depicts a comparison between gene expression at 6 and 18 h. (D) HMOX1 protein levels after 24 h of LPS exposure, determined by WB. Representative blot images are presented (original blots are shown in Figure S7). Data corresponds to the mean ± SEM of four independent experiments and are normalized to the reference gene *Hprt1* (A–C) or three independent experiments and normalized to LPS (D). Statistics: One‐way ANOVA with Tukey's multiple comparisons test and unpaired *t*‐test (*t*). *p* < 0.05 was considered significant. * *p* < 0.05, compared to Ctr; && *p* < 0.01, &&& *p* < 0.001 and &&&& *p* < 0.0001, compared to the control cells of B and S (without LPS); # *p* < 0.05, compared to LPS; *t p* < 0.05 and *tt p* < 0.001.

Since inflammation is deeply associated with oxidative stress, we explored the potential effect of B and S NMW on the antioxidant response of macrophages when challenged with LPS. Therefore, we analyzed *Hmox1* gene expression (by RT‐qPCR) in cells cultured in the different media alone (for 48 h) or exposed to LPS for 6 h (Figure 7A) or 18 h (Figure 7B) and determined Heme Oxygenase 1 (HMOX1) protein levels (by WB) after 24 h of LPS incubation (Figure 7D).

As shown in Figure 7, LPS increased *Hmox1* gene expression at 6 h (Figure 7A) and 18 h (Figure 7B), which was potentiated by both B and S NMW at 6 h, and only by S NMW at 18 h. Indeed, the increase in the expression of *Hmox1* gene seemed to be sustained over time by S NMW, in contrast to what was observed in Ctr and B cell cultures (+LPS 6 h vs. + LPS 18 h; Figure 7C). Similarly, LPS‐induced HMOX1 protein upregulation was also enhanced by B and S NMW (B + LPS and S + LPS, compared to LPS; Figure 7D). These results indicate that LUSO NMW might have an important role in antioxidant pathways triggered by an inflammatory stimulus.

### 3.5 LUSO NMW Enhanced LPS‐Induced RAW 264.7 Macrophage Phagocytic Capacity

**FIGURE 8:**
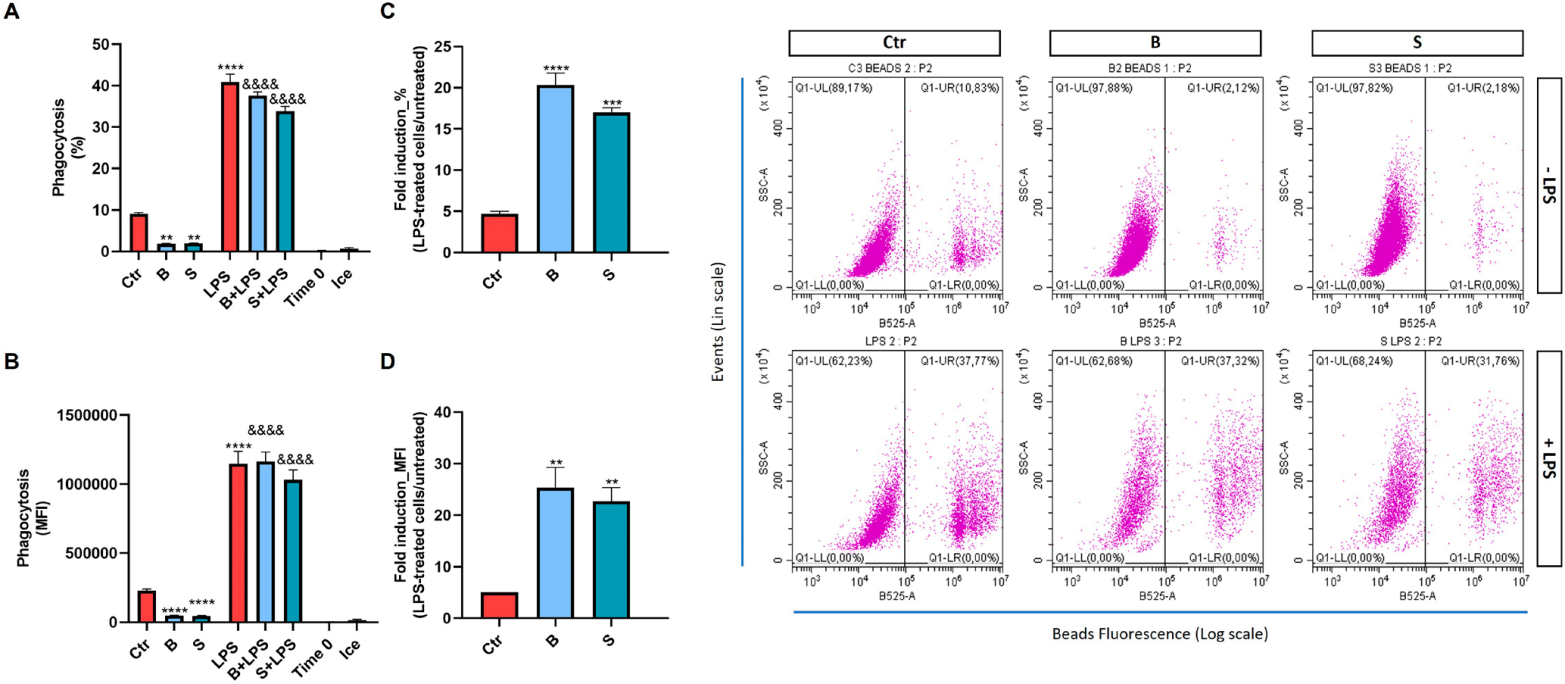
LUSO NMW enhanced LPS‐induced phagocytosis in RAW 264.7 cells. (A–D) Analysis of fluorescent beads internalization (1:10, cell: beads ratio), assessed by flow cytometry. Experiment controls: *Time 0* (the beads were washed after 3 min) and *Ice* (cells placed on ice during beads incubation). Representative dot plots of the experiments are presented. Data correspond to the mean ± SEM of three independent experiments and are represented as % of phagocytic cells (A) and MFI (B). (C, D) Fold induction of LPS‐induced phagocytosis in each experimental condition (Ctr, B, or S). Statistics: One‐way ANOVA with Tukey's multiple comparisons test. *p* < 0.05 was considered significant. ** *p* < 0.01, *** *p* < 0.001 and **** *p* < 0.0001, compared to Ctr; &&&& *p* < 0.0001, compared to the control cells of B and S (without LPS).

Since macrophage activation has been related to an increased phagocytic capacity (de Sousa et al. 2019; Strizova et al. 2023), we investigated the potential of LUSO NMW in boosting macrophage phagocytosis. We used fluorescent amine‐modified polystyrene beads that mimic opsonized cells, which share common routes of degradation with microbial cells, without introducing additional proteins (Erwig et al. 2006). The beads were delivered to macrophages cultured in the different media alone (for 48 h) or exposed to LPS (for 24 h). After 2 h, the % of cells and the MFI were determined by flow cytometry (Figure 8A,B, respectively). Experiment negative controls, *Time 0* (beads delivery for 3 min) and *Ice* (cells placed on ice during beads incubation, which inhibits phagocytosis), are also shown.

The results showed that B and S NMW decreased cell phagocytosis at the basal level, compared to Ctr (Figure 8A,B). However, when challenged with LPS, macrophages exposed to B and S NMW demonstrated similar phagocytic capacity, compared to control cells (B + LPS and S + LPS vs. LPS; Figure 8A,B). Therefore, we determined the fold increase of LPS‐induced phagocytosis in each cell population (by calculating the ratio of LPS stimulated cells/unstimulated cells in each Ctr, B‐ and S‐NMW cell cultures; Figure 8C,D). These results demonstrate that macrophages cultured in B and S NMW respond promptly to LPS, exhibiting enhanced phagocytic affinity and capacity, compared to the cells cultured in regular culture medium (B and S vs. Ctr; Figure 8C,D, respectively).

## 4 Discussion

NMW use in the alleviation and/or treatment of inflammation‐associated diseases is widely recognized, but scientific validation supporting its effects is still insufficient worldwide. Since several natural hot springs are active in Portugal, the present study focused on LUSO NMW, a hyposaline water previously shown to decrease NO and iNOS protein levels in LPS‐activated macrophages (Silva et al. 2020). In the previous study, the pH of culture media diluted in LUSO NMW was maintained acidic (i.e., 5.2, as NMW original pH), since it was focused on the NMW topical use. In the present work, we aimed to explore the anti‐inflammatory effect of LUSO NMW when ingested and shed some light on the biological processes potentially involved. Considering the organism buffer systems to maintain the physiological pH level (Hopkins et al. 2024), the pH of NMW‐containing media was adjusted to 7.2–7.4. In addition, we used LUSO NMW collected directly from the spring and commercially available bottled water for comparison. Macrophages' M1/M2 phenotype, anti‐inflammatory and antioxidant mediators' expression, and phagocytic capacity were evaluated on murine macrophages exposed to LPS (100 ng/mL). Of note, and as far as we know, this is the first study that addresses the impact of NMW on macrophage polarization.

We demonstrated that LUSO NMW increased cells' metabolic capacity at homeostatic conditions and in an inflammation scenario (with LPS; Figure 1). This was not observed in our previous work (Silva et al. 2020), which could be related to the NMW acidic pH. Herein, the increase in cells' metabolism was particularly promoted by B NMW cultures, which could be related to the increase in cell viability (compared to Ctr and S NMW; Figure S8). However, the number of viable cells upon exposure to S NMW was similar to that of Ctr (Figure S8), suggesting that LUSO NMW enhances the metabolic status of macrophages, which only partially reflects cell viability.

At a basal state, LUSO NMW influenced the number of cells that express CD163 (Figure 2C), which was increased only by S NMW (compared to Ctr; Figure 2C), and the phagocytic capacity of macrophages, which was reduced by both B and S NMW (Figure 8A,B), suggesting that LUSO NMW modulates macrophages' phenotype per se. The CD163 scavenger receptor was demonstrated to be associated with an M2 phenotype, indirectly contributing to an anti‐inflammatory response through the uptake and clearance of the oxidative and proinflammatory hemoglobin, leading to the activation of HMOX1 and the production of anti‐inflammatory heme metabolites (Etzerodt and Moestrup 2013). Other reports have demonstrated that CD163 binds and sequesters the TNF‐like weak inducer of apoptosis (TWEAK) protein, involved in the modulation of inflammation (Moreno et al. 2009; Chen et al. 2023).

Controversial studies about macrophages' phagocytic ability have been published, suggesting that it depends on the cells' origin, activation state, and the specific pathogen used. IL‐4 priming (a classical inducer of the M2 phenotype) of mice peritoneal macrophages reduced *N. meningitidis* bacterial phagocytosis, compared to unprimed cells (Varin et al. 2010). In contrast, M2 human macrophages demonstrated to phagocytose more *E. coli* compared to M1 (Schulz et al. 2019; Suleimanov et al. 2024). In RAW 264.7 macrophages, the phagocytic activity was shown to be a characteristic of both M1 and M2 phenotypes (primed by IFN‐γ and IL‐4, respectively), contrary to unprimed cells, although M2 exhibited less phagocytic capacity than M1 macrophages (Lam et al. 2016). Enhanced phagocytosis of bacteria and both non‐opsonized and opsonized latex beads is also associated with murine macrophages' elasticity and adhesion strength, both increased by LPS (McWhorter et al. 2015; Lee et al. 2022). This might be related to increased migration speed, allowing the macrophages to engulf more targets within the same time frame (as reviewed in Lee et al. 2022). Considering the aforementioned, we suggest that LUSO NMW induced unstimulated macrophages to acquire an M2 phenotype since these cells expressed more CD163 and exhibited a lower CD86/CD163 ratio, and showed reduced phagocytic capacity, compared to Ctr. As expected, after being challenged with LPS, all macrophage populations equally increased bead phagocytosis (Figure 8A,B). Cells cultured in LUSO NMW presented a higher fold‐induction in their phagocytic affinity and capacity (Figure 8C,D, respectively), compared to Ctr cells, suggesting that LUSO NMW boosts macrophages' phagocytosis in a pro‐inflammatory environment.

LPS shifted resting macrophages towards M1, as demonstrated by the: (i) reduction in CD206 and increase in CD86 surface markers (Figure 2); (ii) induction of NF‐kB nuclear translocation (Figure 3); (iii) increased iNOS, IL‐1β, IL‐6 and TNF‐α gene expression (Figure 4) and protein levels (Figures 5 and 6); and (iv) increased phagocytic capacity (Figure 8). LUSO NMW significantly reduced LPS‐induced increase of CD86/CD163 (Figure 2H) and CD86/CD206 (Figure 2G) ratio, although the latter did not present statistical significance. In fact, the M2 marker CD206 was not modulated by LUSO NMW (Figure 2A,B), which could be explained by the fact that we did not use additional stimulus (such as IL‐4), usually used to prime M2 macrophages (de Sousa et al. 2019; Strizova et al. 2023). LUSO NMW diminished the expression and release of pro‐inflammatory mediators. It had a profound effect on IL‐6, since it reduced *Il6* gene expression at 6 h (peak of inflammation), at 18 h (pre‐resolution phase) (Figure 4C,G, respectively), and the secreted levels of IL‐6 (Figure 6C) after 24 h of LPS exposure that mimics the resolution phase of the inflammatory process. We should notice that the effect of S NMW was less pronounced, suggesting that S NMW could take a longer time to exert its effect. LPS‐induced IL‐1β supernatant levels and TNF‐α secretion were also reverted by LUSO NMW, probably through different mechanisms. While B NMW significantly inhibited IL‐1β supernatant levels, S NMW only induced a non‐significant reduction (Figure 6B). In contrast, the decrease in TNF‐α secreted levels by LUSO NMW was significantly achieved by S NMW (Figure 6D). In this work, the upstream pathways of protein processing and activation have not been explored. However, the influence of NMW on pro‐inflammatory enzymes has been described (Aversano et al. 2020), and the effect of LUSO NMW on cleaving enzymes, such as caspase‐1 (or extracellular proteases in RAW 264.7 cellular model) and tumor necrosis factor‐alpha converting enzyme (TACE), responsible for the production and release of mature IL‐1β and TNF‐α, respectively, should be a matter of further investigation. Intriguingly, iNOS and NO were not affected by LUSO NMW, which apparently contradicts our previous results (Silva et al. 2020). As mentioned, in the present work, we adjusted the pH of NMW‐containing medium to 7.2, which is the optimal pH for NO production by iNOS (Huang et al. 2002). Interestingly, Bystrom et al. (2008) demonstrated sustained iNOS expression in another macrophage subtype associated with the resolution phase of inflammation (Bystrom et al. 2008). The authors used peritoneal macrophages derived from mice injected intraperitoneally with a low and high dose of zymosan, to mimic mild and transient inflammation resulting in full recovery, and a progressive and prolonged response leading to systemic inflammation, respectively. Bystrom and colleagues showed that resolving inflammation promotes a macrophage hybrid phenotype that is neither classically nor alternatively activated, denominated resolution phase macrophages (rMs). These macrophages expressed high levels of COX 2, iNOS, IL‐10, and intracellular cAMP, and exhibited less capacity for clearing bacteria, compared to M1 macrophages derived from the non‐resolving model. The authors further suggested that they are essential for restoring tissue homeostasis and combating future infections (Bystrom et al. 2008).

LUSO NMW increased HMOX1 gene expression and protein levels (Figure 7), and S NMW induced an increase in *Sod2* mRNA levels (Figure S9D–F), which encodes the mitochondrial superoxide dismutase enzyme, suggesting that LUSO NMW might also activate antioxidant‐related pathways. However, additional proteins should be investigated to undoubtedly support LUSO NMW's antioxidant properties.

It has been demonstrated that HMOX1‐increased expression can be mediated by multiple signaling pathways, driving the phenotypic shift to M2 macrophages (Lee and Chau 2002; Sierra‐Filardi et al. 2010; Naito et al. 2014), and several reports have shown an interrelation between HMOX1 expression and IL‐10 signaling. IL‐10 is an anti‐inflammatory cytokine involved in the inhibition of cytokine production by macrophages (e.g., IL‐1β, IL‐6, IL‐8, IL‐12, TNF‐α). In murine macrophages, IL‐10 induced the expression of HMOX1 via p38 MAPK‐dependent pathway, and HMOX1 inhibition prevented IL‐10 from reducing LPS‐induced TNF‐α production (Lee and Chau 2002). Moreover, IL10 secretion is also elicited after CD163 binding of hemoglobin–haptoglobin complexes, inducing HMOX1 (Philippidis et al. 2004). In Sierra‐Filardi et al. (2010) work, the CD163‐IL‐10‐HMOX1 axis has been proposed as a relevant path in the anti‐inflammatory and immunosuppressive activity of M2‐polarized macrophages, derived from human peripheral blood mononuclear cells (Sierra‐Filardi et al. 2010). In our study, we observed an increase in *Il10* mRNA levels in LPS‐exposed macrophages (Figure S9A–C), which were reduced by B NMW after 18 h of LPS incubation (Figure S9B). On the contrary, S NMW sustained *Il10* mRNA over time (18 h compared to 6 h; Figure S9C). We did not determine IL‐10 secretion levels, but regarding these results and the slight increase in *Il10* gene expression of macrophages exposed to S NMW (compared to LPS), the cells may release more IL‐10, due to HMOX1 activation by LUSO NMW (more evident in S NMW‐exposed cells), as reported by Sierra‐Filardi et al. (2010). Also aligning with this hypothesis is the reduction of TNF‐α (protein secretion at 24 h; Figure 6D) in S NMW‐exposed cultures, which might be promoted by IL‐10 as demonstrated elsewhere (Lee and Chau 2002).

Regarding that Type I ultrapure water used to make cell culture medium has an osmolarity and osmolality of 0 mOsm/L and 0 mOsm/kg, and that LUSO NMW displays similar values (osmolality ~5 mOsm/Kg) (Silva et al. 2020), we do not attribute its effects to these parameters. In addition, since we adjusted all culture media to the physiologic pH (pH 7.2), we also disregarded its effect. Thus, it is plausible to conclude that LUSO NMW's anti‐inflammatory properties are due mainly to its mineral composition. However, relating the outcomes observed in macrophages exposed to LUSO NMW with its mineral content is a difficult task and has not been investigated in this work. Nevertheless, we can hypothesize that the supplementation of regular culture media with the minerals present in LUSO NMW contributes to the differences observed between Ctr and LUSO NMW‐cultured macrophages. Even though it has low mineralization, LUSO NMW surely contributes to an increased mineral concentration (e.g., sodium, magnesium, potassium, and calcium) already present in DMEM, and adds fundamental trace elements (e.g., zinc and copper), which are absent in cell culture medium. Minerals such as magnesium and the fundamental trace element zinc have essential roles in enzymatic reactions, mitochondrial function, DNA and protein synthesis, and gene expression. Both are involved and contribute to antioxidant defense mechanisms, proinflammatory and anti‐inflammatory cytokines balance, and immune system regulation (the role of minerals in health with a particular focus on the immune system has been extensively reviewed) (Quattrini et al. 2016; Weyh et al. 2022; Stefanache et al. 2023). Interestingly, recent reports have investigated the influence of magnesium in macrophage differentiation, where it was demonstrated that high intracellular magnesium concentration induced the switch from M1 to an M2‐like macrophage phenotype, in murine (i.e., RAW 264.7) (Li et al. 2018; Zhao et al. 2022) and human macrophages (i.e., THP‐1‐derived macrophages) (Oh et al. 2023). The major differences detected between the cells cultured in S and B NMW were related to gene expression, after 18 h of LPS exposure. S NMW‐exposed macrophages showed reduced expression of *Tnfa* and increased expression of *Sod2* and *Il10*, compared with B NMW‐cultured cells. Micronutrients are capable of modulating gene transcription and translation directly by interacting with the genome (via *nutrient response elements*) and indirectly by impacting several mechanisms, including DNA methylation, histone modification, and microRNA (miRNA) integrity (Beckett et al. 2014). B and S NMW have a similar mineral composition (Table S1), but the chemical analysis was performed before bottling, and alterations in NMW composition due to plastic contaminants and storage‐induced chemical release, from the bottle to the water, might occur (Quattrini et al. 2016). Moreover, we did not have access to a detailed chemical analysis report of bottled LUSO NMW, particularly concerning the presence and concentration of trace elements, hindering any assumptions about mineral content and gene expression correlation. Importantly, although LUSO NMW microbiological analysis confirmed the absence of pathogenic bacteria (including total coliforms, fecal coliforms such as *Escherichia coli* , fecal *Streptococcus*, and *Pseudomonas aeruginosa* ), we cannot exclude the presence of other bacteria and/or metabolites of bacterial origin, which were not determined and may have significant effects on inflammation and immune modulation. In fact, it has been shown that beneficial microorganisms can produce bioactive compounds with anti‐inflammatory properties, which may help reduce inflammatory responses (Biagi et al. 2010; Lazar et al. 2018; Aversano et al. 2020). This, and the lack of studies addressing NMW effect on macrophage polarization and function, hampered the discussion of our results.

Nevertheless, in our experimental conditions, LUSO NMW promoted an anti‐inflammatory phenotype (decreased CD86/CD163 ratio), decreased pro‐inflammatory parameters (i.e., *Il6* gene expression; IL‐1β supernatant levels, and IL‐6 and TNF‐α secretion levels), increased the antioxidant enzyme HMOX1 (i.e., gene expression and protein levels), and enhanced LPS‐induced phagocytic capacity. Of note, in preliminary experiments using human macrophages, exposure to S NMW (which showed more robust results regarding RAW 264.7 macrophage polarization and potential antioxidant effects, compared to B NMW), also seemed to promote an M2 anti‐inflammatory phenotype (Figures S10 and S11). We used THP‐1‐derived macrophages induced by a low concentration of Phorbol 12‐myristate 13‐acetate (PMA; 5 ng/mL) (Park et al. 2007; Baxter et al. 2020) for 24 h, as previously described by Baxter et al. (2020). Upon PMA treatment, the cells become adherent and with protrusions (Figure S10A, red arrows), indicative of macrophage differentiation. After a 72 h resting period in fresh medium (without PMA), we observed that the mRNA levels of the M1 marker genes, *IRF1*, *STAT1* and *CCR7*, and of *IL1B* were reduced in macrophages cultured in S NMW (S + PMA, compared to PMA; Figure S10B). *MRC1* (CD206) gene expression also seemed to be decreased by S NMW, likely resembling the trend observed in RAW 264.7 macrophages (S + LPS, compared to LPS; Figure 2B, MFI). In contrast, the M2 marker gene *CD163*, as well as *HMOX1*, were clearly augmented (S + PMA, compared to PMA; Figure S10B), which aligns with the results obtained in RAW 264.7 cells (Figures 2 and 7, respectively). Moreover, in primary human macrophages (CD14+ monocytes differentiated with granulocyte‐macrophage colony‐stimulating factor, GM‐CSF), exposed to an inflammatory cocktail (LPS, INF‐γ and TNF‐α) for 48 h, flow cytometry analysis revealed a higher % of CD163‐expressing cells in S NMW cultures (S + Cocktail vs Cocktail; Figure S11A), highlighting the potential modulation of CD163 expression by S LUSO NMW (as evaluated by flow cytometry and gene expression analysis in different experimental models).

Overall, these results validate the anti‐inflammatory effect of LUSO NMW, by shifting M1 macrophages towards an M2 phenotype, without any additional stimulus (e.g., IL4, IL10 or IL13), but further experiments are needed to undoubtedly infer about the broad spectrum of M2 subsets.

Some limitations to this study must be drawn. RAW 264.7 is a well‐established macrophage cell line with stable phenotypic and functional features. They exhibit basic macrophage‐like functions (e.g., phagocytosis and pinocytosis) and are widely used for studying host‐pathogen interactions and immune responses in vitro. In addition, these cells display an M0 phenotype in their basal state and a notable capacity to polarize into M1 or M2 phenotypes. However, RAW 264.7 are transformed cells, and may not truly represent primary macrophages' features (Taciak et al. 2018). Moreover, there are considerable physiological and immunological differences between mice and humans, including in the murine M1 or M2 signature genes and in the expression of M1 and M2 surface markers. Therefore, these pitfalls must be considered during data interpretation (Schneemann and Schoeden 2007; Tarique et al. 2015), though our preliminary results obtained with human macrophages appear to be consistent with those obtained from RAW 264.7 cells. Additionally, the optimal NMW concentration under in vitro conditions should consider its dilution within the organism, as well as its sustained intake over an extended period, to better replicate human consumption patterns. However, accurately reproducing these conditions in vitro is challenging, particularly due to the difficulty of maintaining stable experimental parameters over time (e.g., cell passage number and viability). Although acute exposure does not fully reflect chronic intake, we opted to expose cells to the maximum concentration over a shorter timeframe, as previously reported by us (Silva et al. 2020) and others (Nam et al. 2020), to draw inferences regarding the potential effects of LUSO NMW on human health.

In conclusion, certain mechanisms identified under these in vitro conditions are difficult to investigate in humans, underscoring the value of these models for elucidating cellular responses. Nevertheless, LUSO NMW therapeutic effects should be deeply explored, including as an adjuvant in immune‐ and anti‐inflammatory‐based therapies, using accurate cells as primary human‐derived macrophages, with well‐designed experimental conditions keeping in mind important factors that might influence cell responses. Furthermore, comparative studies involving different types of NMW should be conducted, incorporating a thorough and up‐to‐date analysis of their mineral content and microbiota.

## Author Contributions

**Beatriz Rodrigues:** investigation. **Mylène Carrascal:** investigation. **Rosa Resende:** writing – review and editing, investigation. **Rui Soares:** funding acquisition, resources, data curation. **Ana Silva:** funding acquisition, investigation, writing – original draft, methodology, resources, data curation, formal analysis, project administration, conceptualization. **Marisa Azul:** writing – review and editing. **Cláudia Cid:** investigation. **Maria do Céu Sousa:** writing – review and editing. **Conceição Egas:** writing – review and editing. **Cláudia F. Pereira:** writing – review and editing. **Maria Teresa Cruz:** data curation, validation.

## Funding

This work was supported by [COMPETE 2020–Operational Programme for Competitiveness and Internationalization and FCT–Fundação para a Ciência e a Tecnologia] (Grant numbers [UIDB/04539/2020], [UIDP/04539/2020], and [LA/P/0058/2020]) and [Sociedade Central de Cervejas].

## Conflicts of Interest

The present study was financed by Sociedade Central de Cervejas, owner of LUSO's brand, and the author Rui Soares (MD) is the clinical Director of LUSO's Thermal SPA. The authors declare that this has not influenced the results and the conclusion of this work.

## Supporting information


**Figure S1:** M1 and M2 phenotype assessed by flow cytometry.
**Figure S2:** NF‐kB nuclear translocation.
**Figure S3:** Housekeeping gene expression.
**Figure S4:** Phagocytic capacity assessed by flow cytometry.
**Figure S5:** Ik‐Bα phosphorylation levels.
**Figure S6:** Pro‐inflammatory protein levels.
**Figure S7:** Original blot membranes depicted in the manuscript.
**Figure S8:** Cellular viability (Trypan Blue dye exclusion assay).
**Figure S9:** fsn371962‐sup‐0001‐Supinfo.pptx. *Il10* and *Sod2* gene expression.
**Figure S10:** M1 and M2 marker genes in THP‐1‐derived macrophages exposed to S LUSO NMW.
**Figure S11:** Expression of the M2 marker CD163 and M1 marker CD86 in human macrophages exposed to S LUSO NMW.
**Table S1:** Luso NMW physicochemical composition according to the certified analytical reports of 2023.
**Table S2:** List of primers.

## Data Availability

The data that support the findings of this study are available from the corresponding author upon reasonable request.
