# Supplementary material for: Hyposaline LUSO Mineral Water Drives Murine Macrophage Polarization Towards an Anti‐Inflammatory M2 Phenotype
Source: Food Sci Nutr. 2026 Jul 8;14(7):e71962. doi: 10.1002/fsn3.71962 (PMC13343306; doi:10.1002/fsn3.71962)
Supplement: Supplementary file 1 — Figure S1: M1 and M2 phenotype assessed by flow cytometry. Figure S2: NF‐kB nuclear translocation. Figure S3: Housekeeping gene expression. Figure S4: Phagocytic capacity assessed by flow cytometry. Figure S5: Ik‐Bα phosphorylation levels. Figure S6: Pro‐inflammatory protein levels. Figure S7: Original blot membranes depicted in the manuscript. Figure S8: Cellular viability (Trypan Blue dye exclusion assay). Figure S9: fsn371962‐sup‐0001‐Supinfo.pptx. Il10 and Sod2 gene expression. Figure S10: M1 and M2 marker genes in THP‐1‐derived macrophages exposed to S LUSO NMW. Figure S11: Expression of the M2 marker CD163 and M1 marker CD86 in human macrophages exposed to S LUSO NMW. Table S1: Luso NMW physicochemical composition according to the certified analytical reports of 2023. Table S2: List of primers. [file FSN3-14-e71962-s001.pptx]

## Slide 1
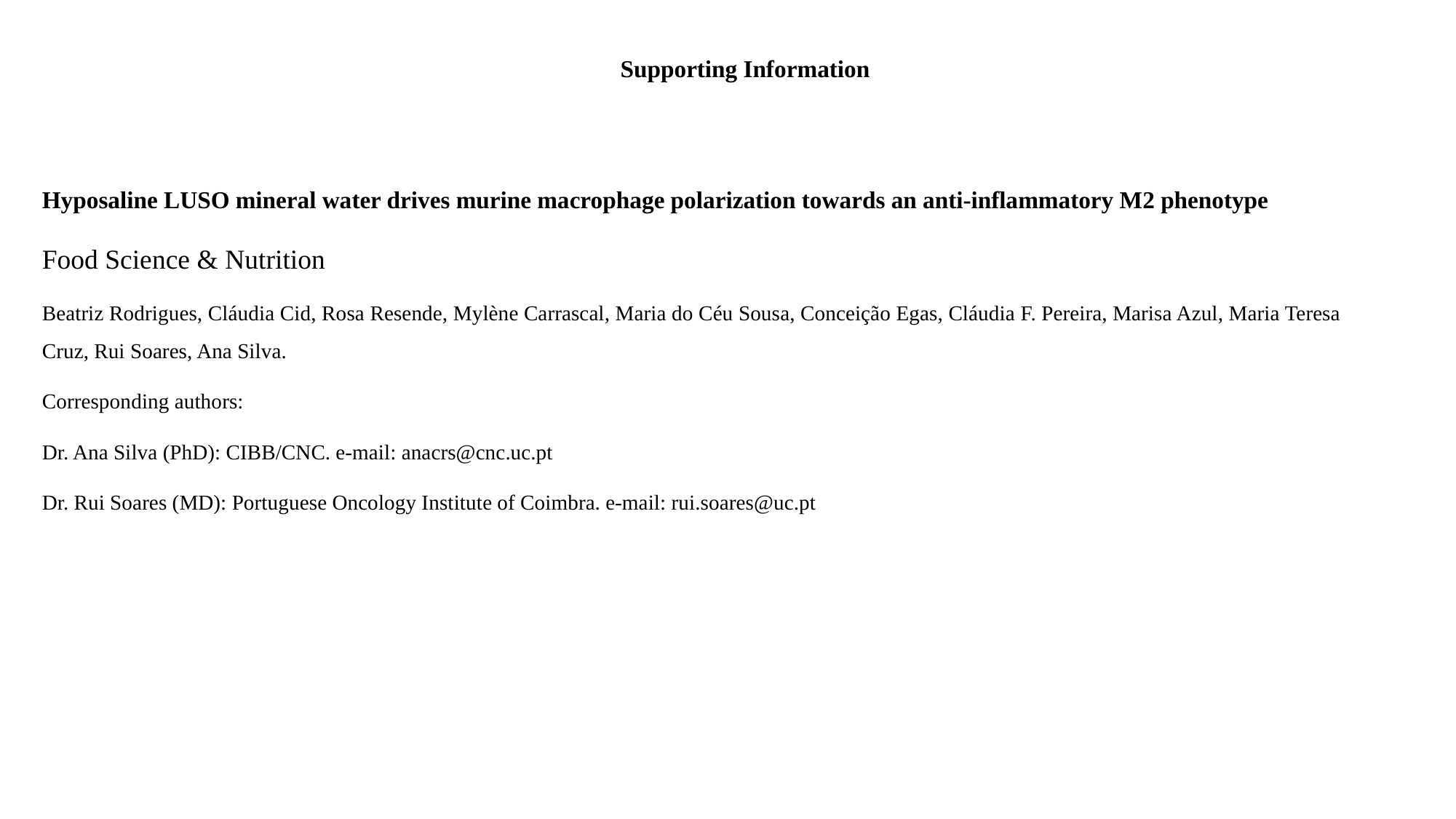

Supporting Information
Hyposaline LUSO mineral water drives murine macrophage polarization towards an anti-inflammatory M2 phenotype
Food Science & Nutrition
Beatriz Rodrigues, Cláudia Cid, Rosa Resende, Mylène Carrascal, Maria do Céu Sousa, Conceição Egas, Cláudia F. Pereira, Marisa Azul, Maria Teresa Cruz, Rui Soares, Ana Silva.
Corresponding authors:
Dr. Ana Silva (PhD): CIBB/CNC. e-mail: anacrs@cnc.uc.pt
Dr. Rui Soares (MD): Portuguese Oncology Institute of Coimbra. e-mail: rui.soares@uc.pt

## Slide 2
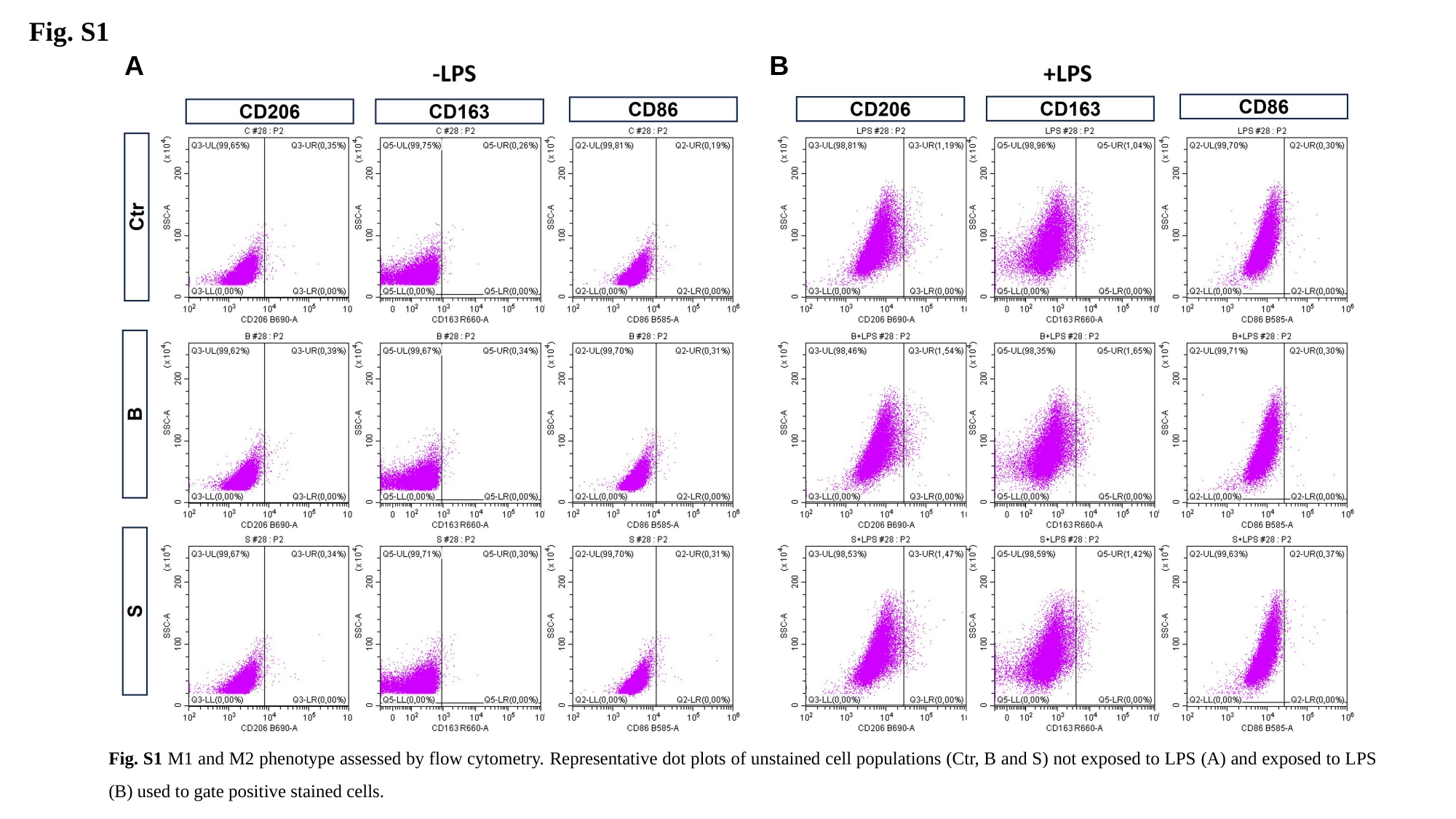

Fig. S1
A
B
Fig. S1 M1 and M2 phenotype assessed by flow cytometry. Representative dot plots of unstained cell populations (Ctr, B and S) not exposed to LPS (A) and exposed to LPS (B) used to gate positive stained cells.

## Slide 3
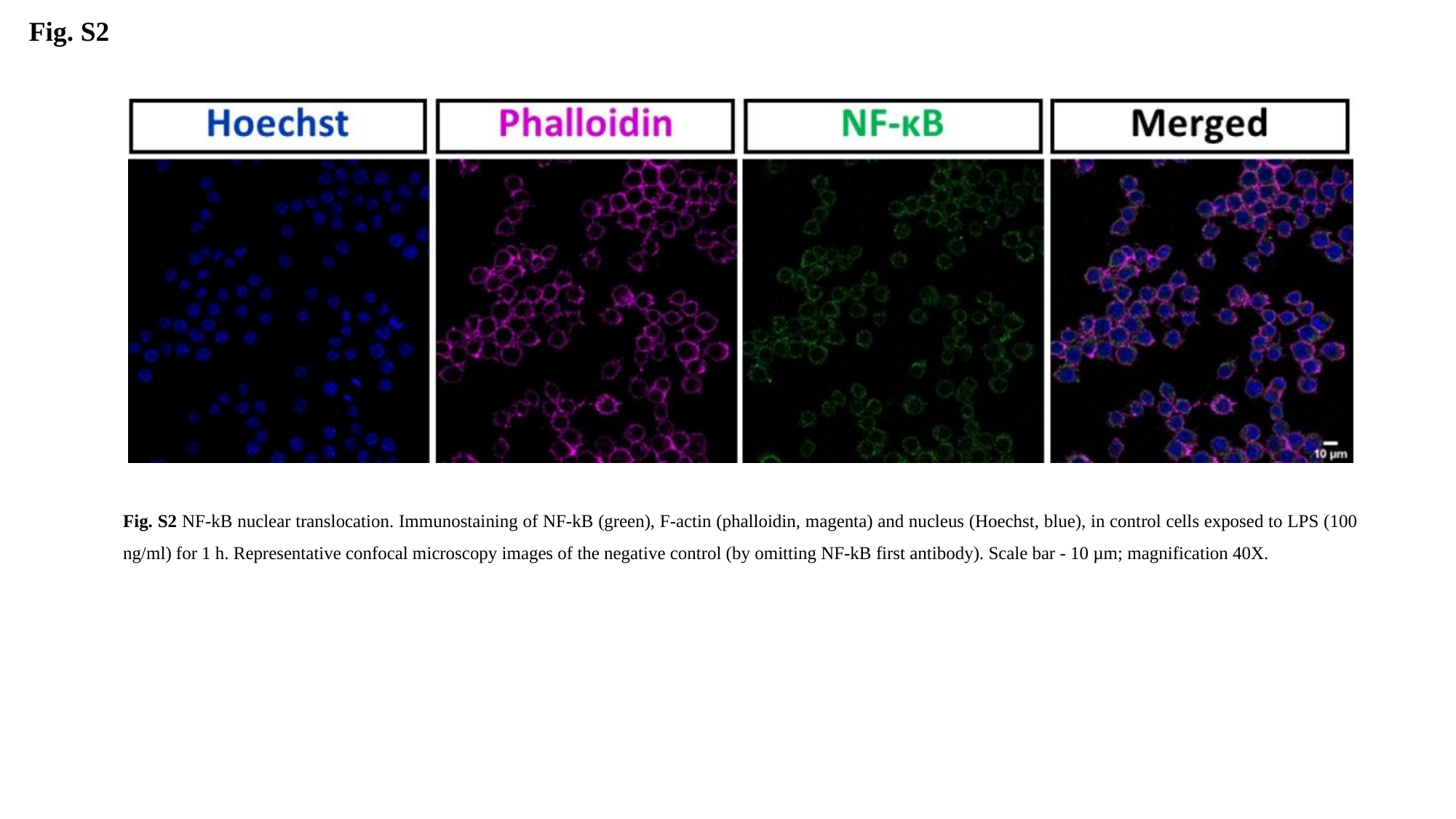

Fig. S2
Fig. S2 NF-kB nuclear translocation. Immunostaining of NF-kB (green), F-actin (phalloidin, magenta) and nucleus (Hoechst, blue), in control cells exposed to LPS (100 ng/ml) for 1 h. Representative confocal microscopy images of the negative control (by omitting NF-kB first antibody). Scale bar - 10 µm; magnification 40X.

## Slide 4
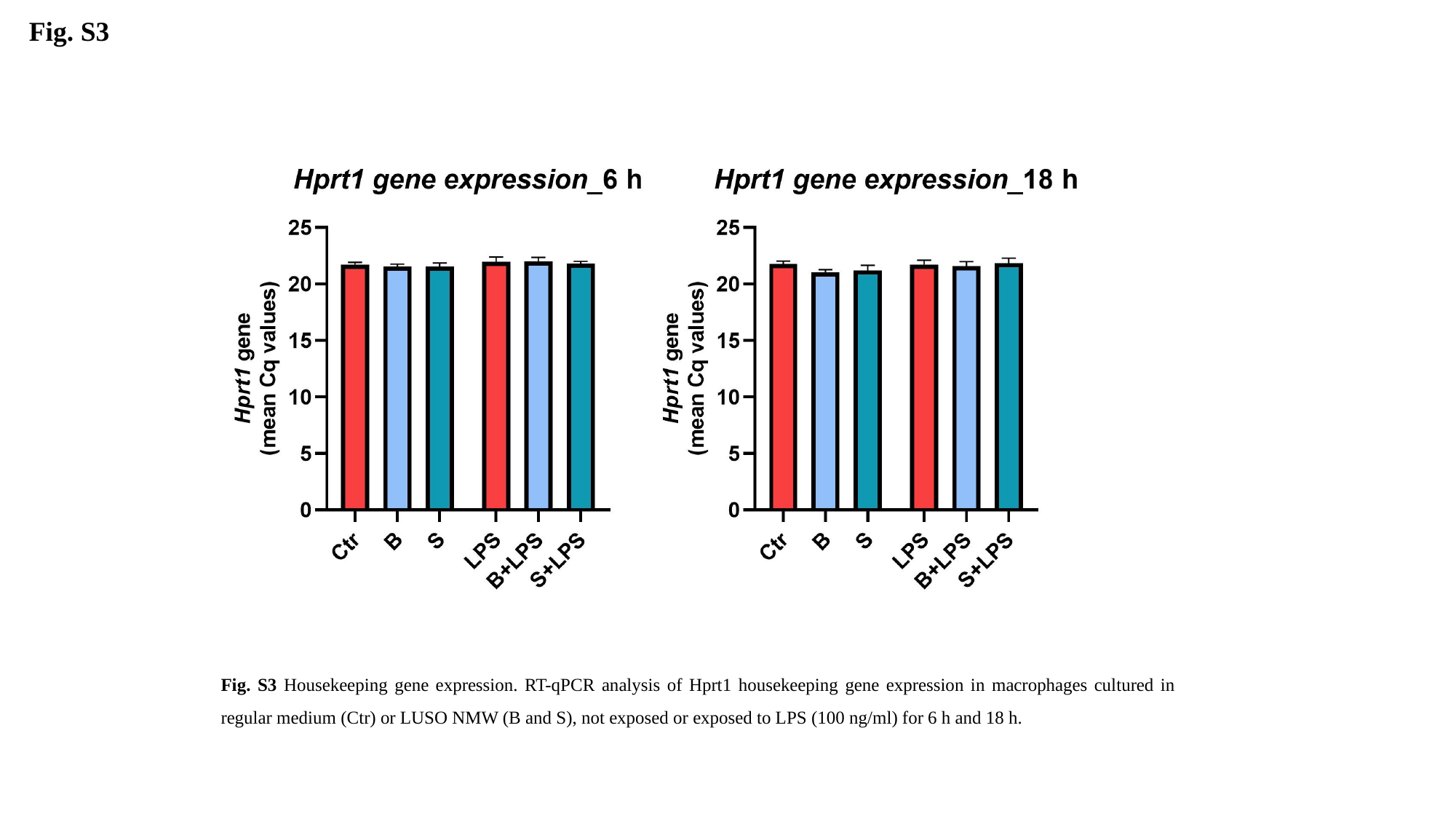

Fig. S3
Fig. S3 Housekeeping gene expression. RT-qPCR analysis of Hprt1 housekeeping gene expression in macrophages cultured in regular medium (Ctr) or LUSO NMW (B and S), not exposed or exposed to LPS (100 ng/ml) for 6 h and 18 h.

## Slide 5
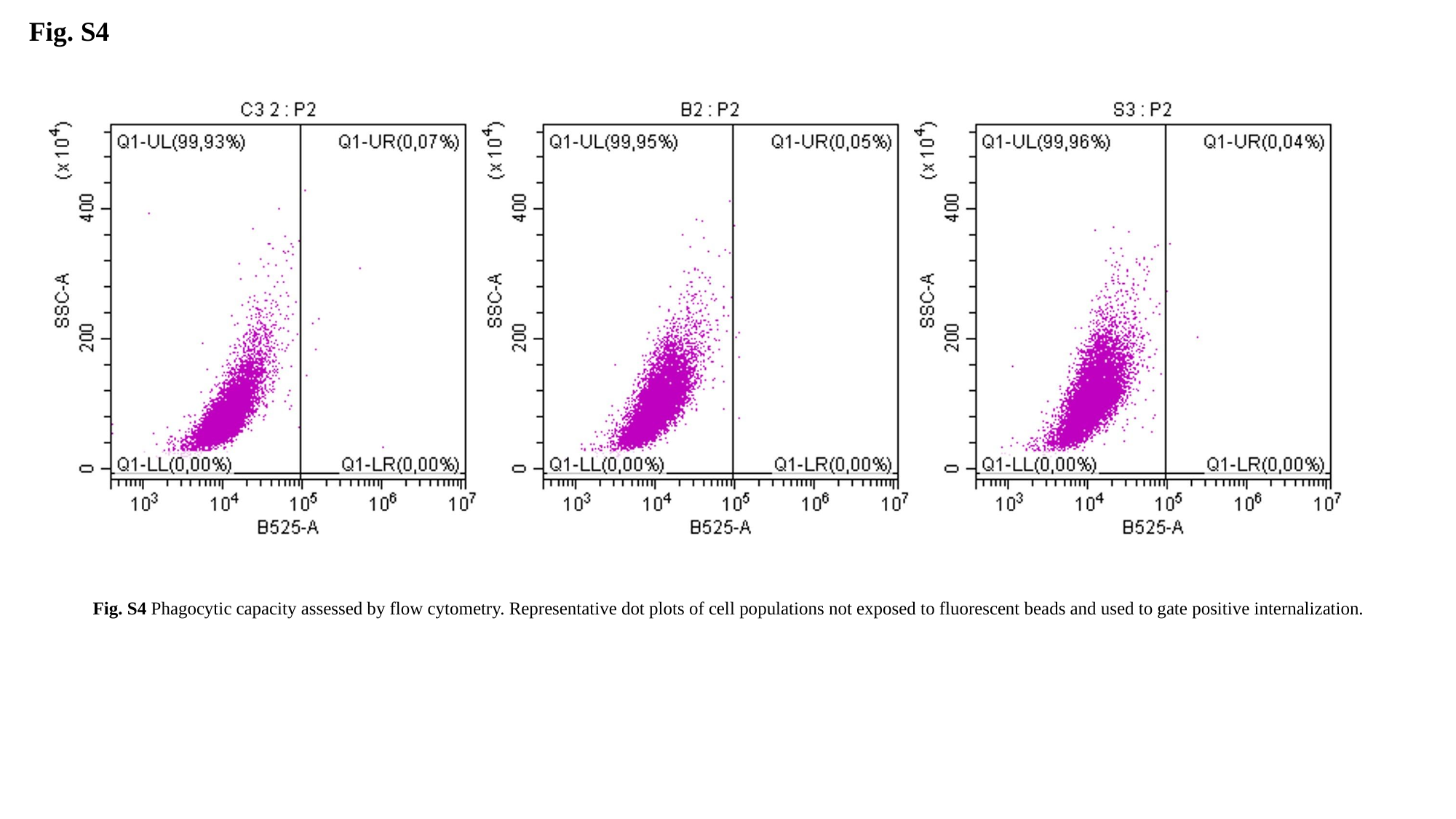

Fig. S4
Fig. S4 Phagocytic capacity assessed by flow cytometry. Representative dot plots of cell populations not exposed to fluorescent beads and used to gate positive internalization.

## Slide 6
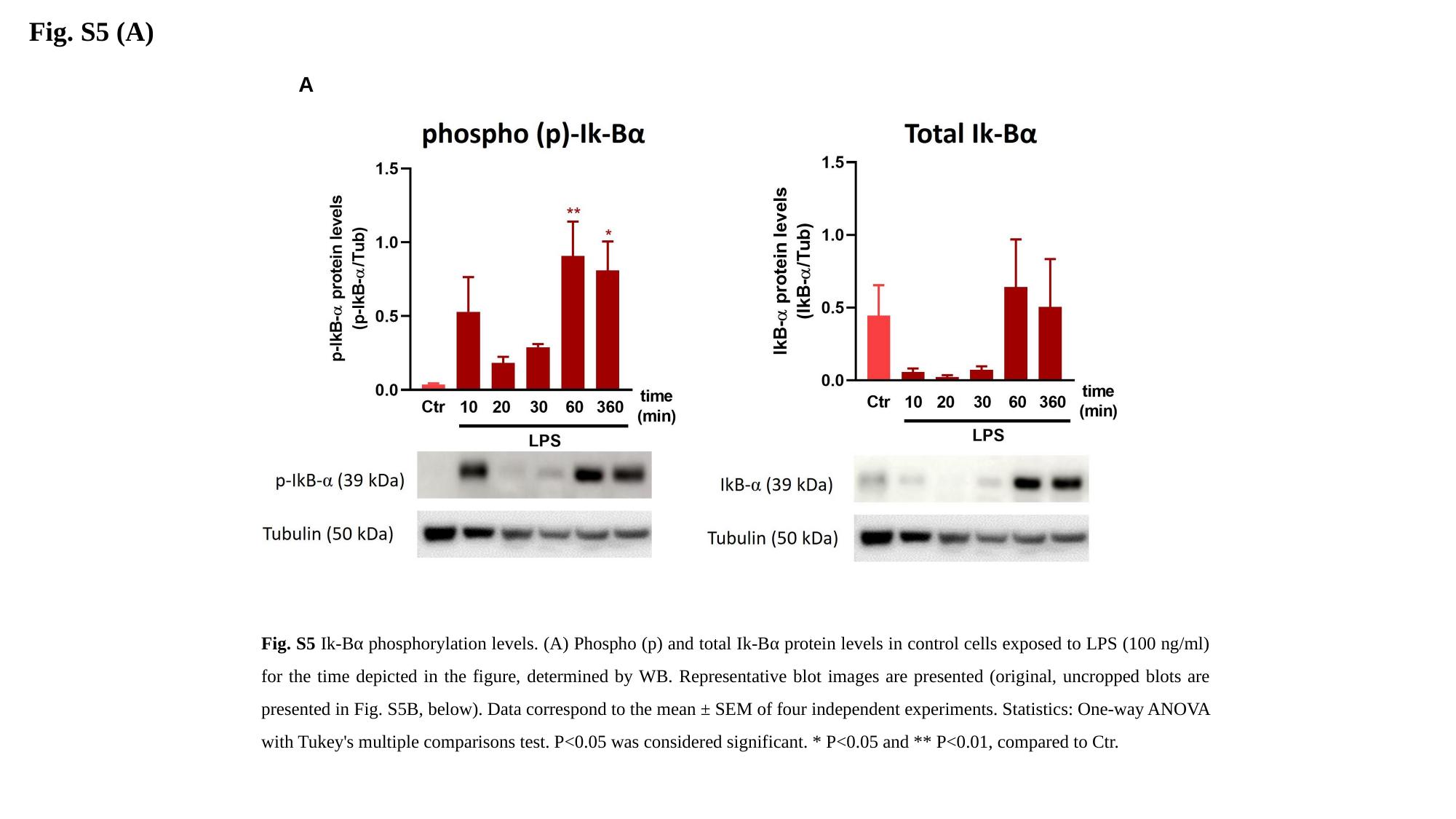

Fig. S5 (A)
A
Fig. S5 Ik-Bα phosphorylation levels. (A) Phospho (p) and total Ik-Bα protein levels in control cells exposed to LPS (100 ng/ml) for the time depicted in the figure, determined by WB. Representative blot images are presented (original, uncropped blots are presented in Fig. S5B, below). Data correspond to the mean ± SEM of four independent experiments. Statistics: One-way ANOVA with Tukey's multiple comparisons test. P<0.05 was considered significant. * P<0.05 and ** P<0.01, compared to Ctr.

## Slide 7
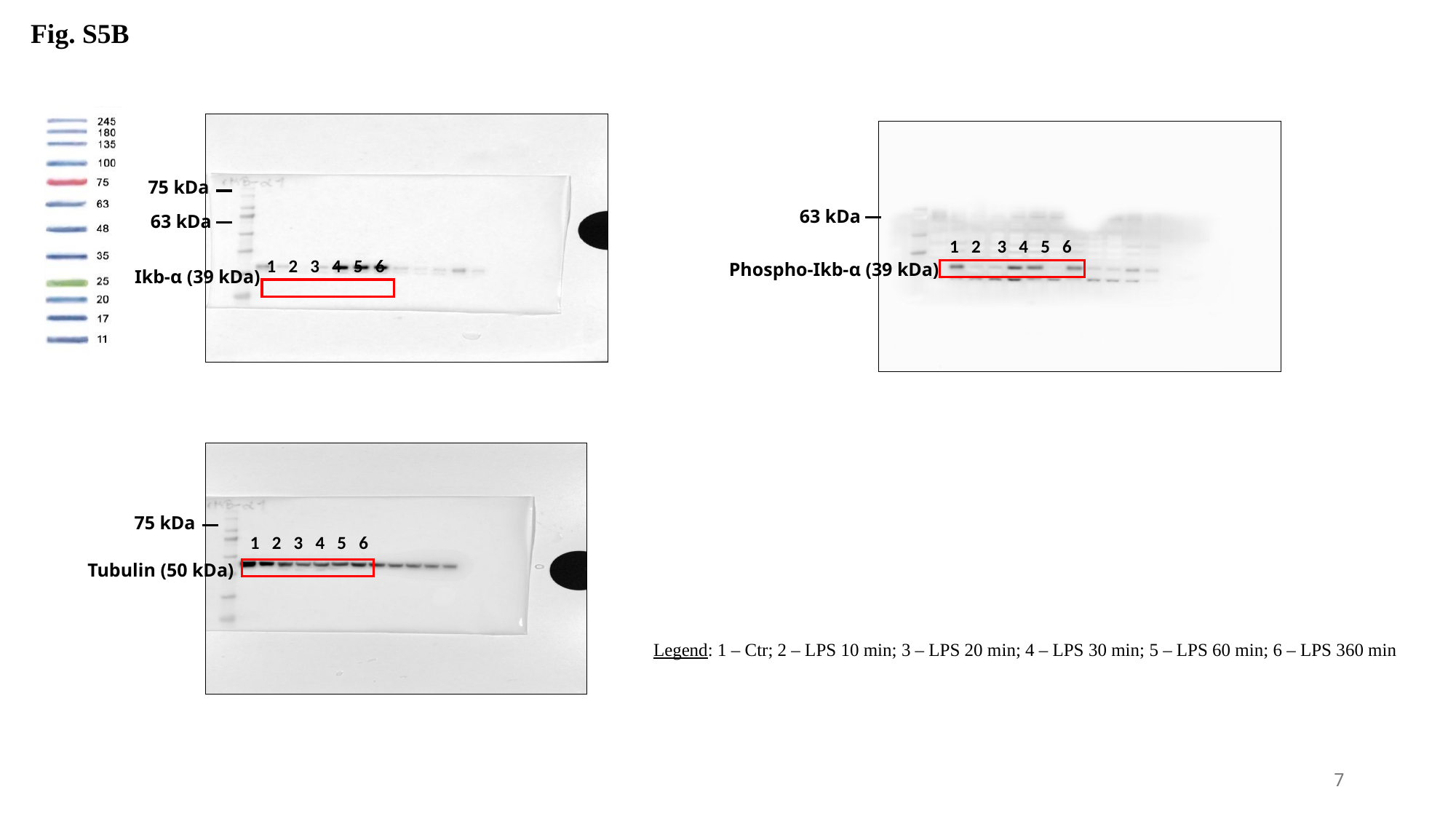

Fig. S5B
1 2 3 4 5 6
1 2 3 4 5 6
75 kDa
63 kDa
63 kDa
Phospho-Ikb-α (39 kDa)
Ikb-α (39 kDa)
1 2 3 4 5 6
75 kDa
Tubulin (50 kDa)
Legend: 1 – Ctr; 2 – LPS 10 min; 3 – LPS 20 min; 4 – LPS 30 min; 5 – LPS 60 min; 6 – LPS 360 min
7

## Slide 8
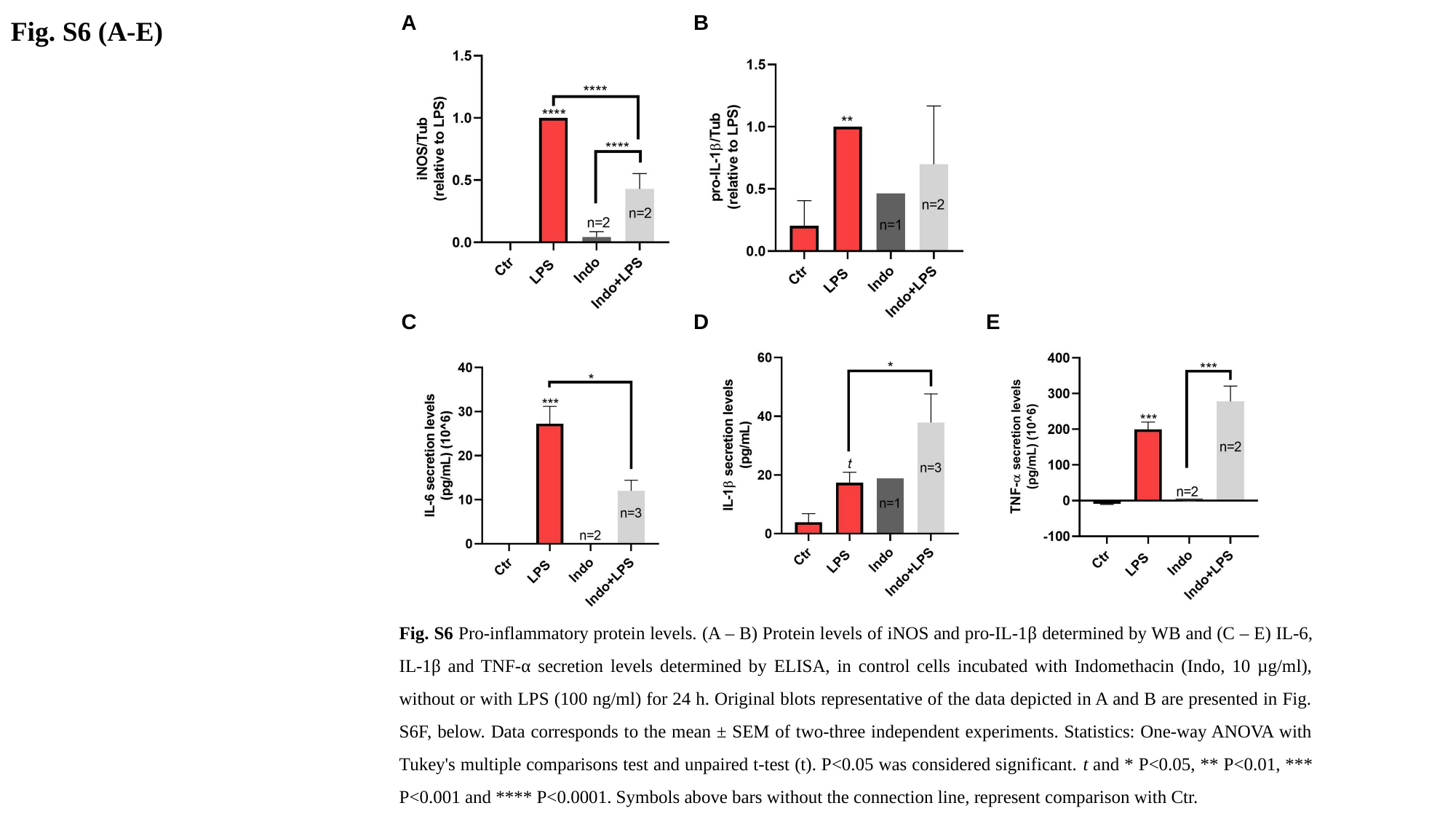

A
B
Fig. S6 (A-E)
C
D
E
Fig. S6 Pro-inflammatory protein levels. (A – B) Protein levels of iNOS and pro-IL-1β determined by WB and (C – E) IL-6, IL-1β and TNF-α secretion levels determined by ELISA, in control cells incubated with Indomethacin (Indo, 10 µg/ml), without or with LPS (100 ng/ml) for 24 h. Original blots representative of the data depicted in A and B are presented in Fig. S6F, below. Data corresponds to the mean ± SEM of two-three independent experiments. Statistics: One-way ANOVA with Tukey's multiple comparisons test and unpaired t-test (t). P<0.05 was considered significant. t and * P<0.05, ** P<0.01, *** P<0.001 and **** P<0.0001. Symbols above bars without the connection line, represent comparison with Ctr.

## Slide 9
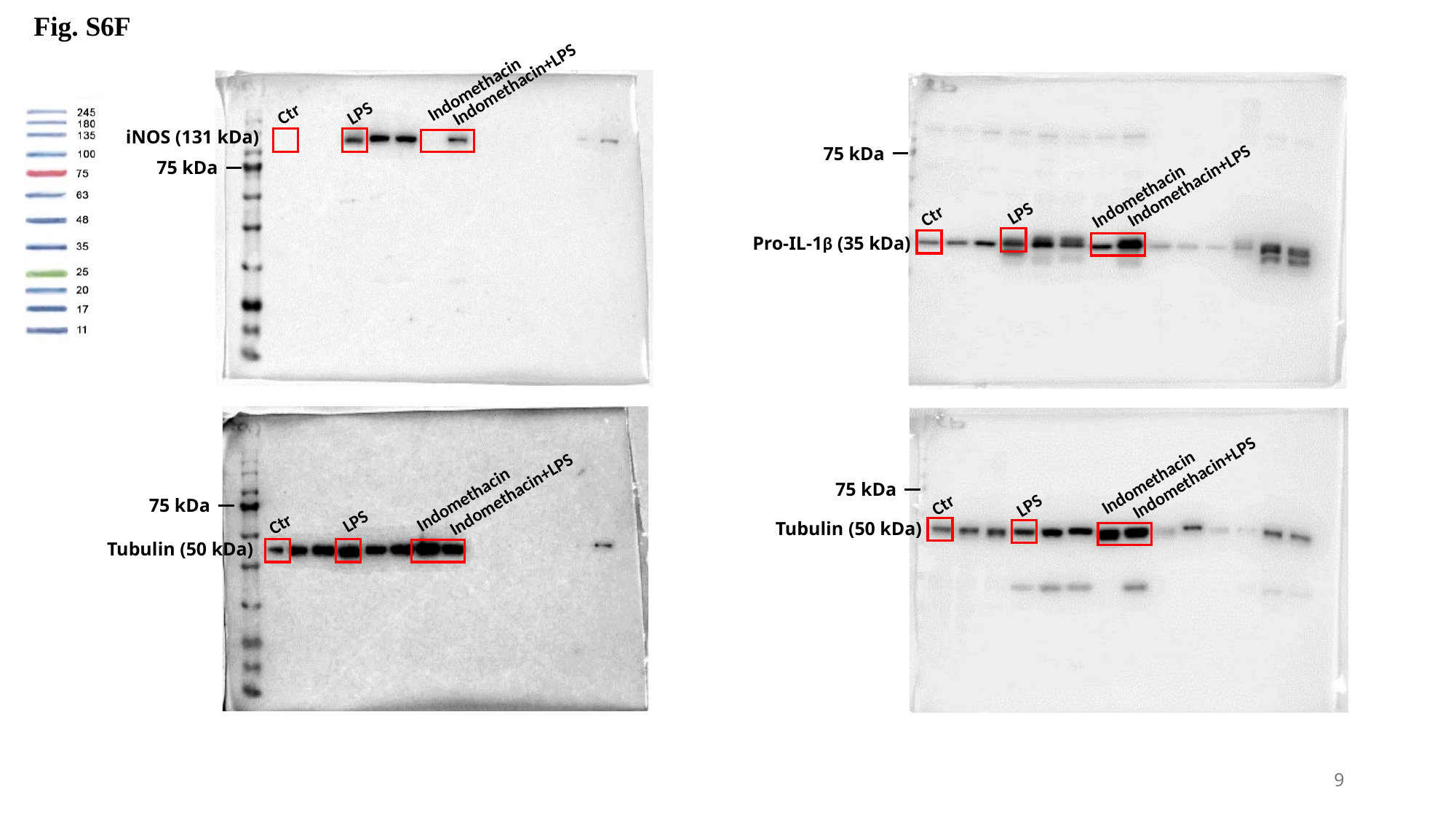

Fig. S6F
Indomethacin+LPS
Indomethacin
LPS
Ctr
iNOS (131 kDa)
75 kDa
75 kDa
Indomethacin+LPS
Indomethacin
LPS
Ctr
Pro-IL-1β (35 kDa)
Indomethacin+LPS
Indomethacin
Indomethacin+LPS
75 kDa
Indomethacin
Ctr
LPS
75 kDa
LPS
Ctr
Tubulin (50 kDa)
Tubulin (50 kDa)
9

## Slide 10
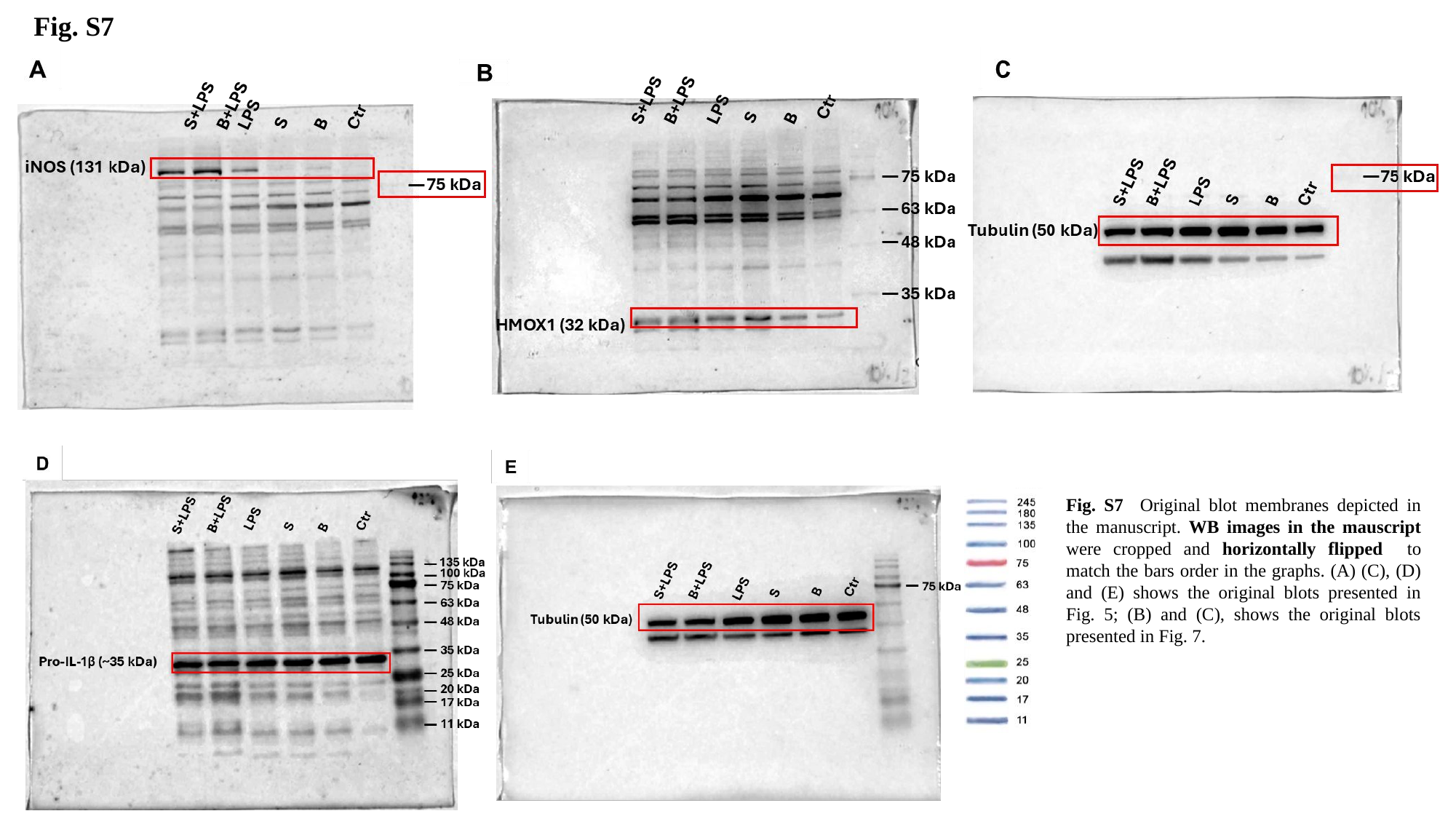

Fig. S7
Fig. S7 Original blot membranes depicted in the manuscript. WB images in the mauscript were cropped and horizontally flipped to match the bars order in the graphs. (A) (C), (D) and (E) shows the original blots presented in Fig. 5; (B) and (C), shows the original blots presented in Fig. 7.

## Slide 11
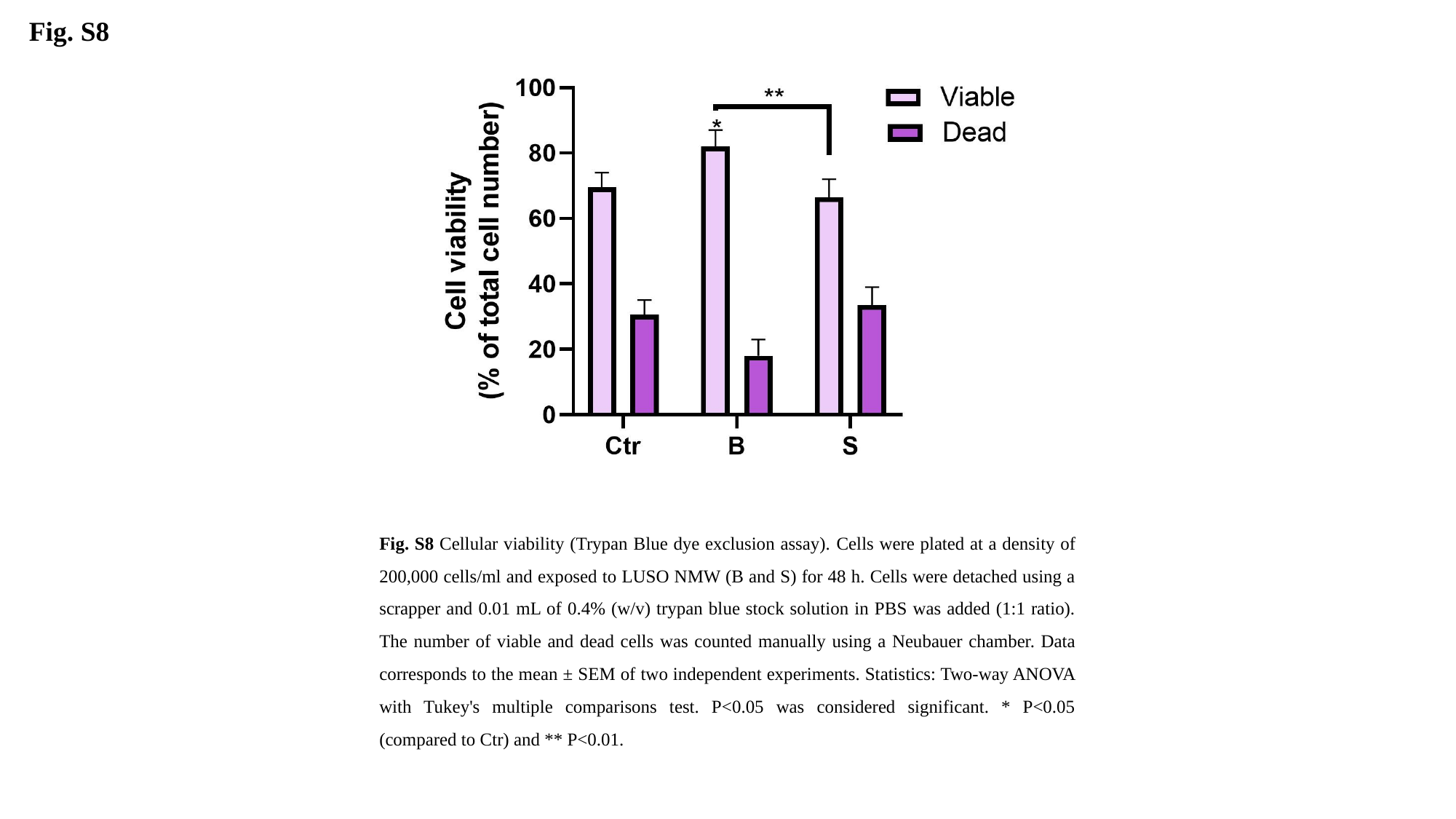

Fig. S8
Fig. S8 Cellular viability (Trypan Blue dye exclusion assay). Cells were plated at a density of 200,000 cells/ml and exposed to LUSO NMW (B and S) for 48 h. Cells were detached using a scrapper and 0.01 mL of 0.4% (w/v) trypan blue stock solution in PBS was added (1:1 ratio). The number of viable and dead cells was counted manually using a Neubauer chamber. Data corresponds to the mean ± SEM of two independent experiments. Statistics: Two-way ANOVA with Tukey's multiple comparisons test. P<0.05 was considered significant. * P<0.05 (compared to Ctr) and ** P<0.01.

## Slide 12
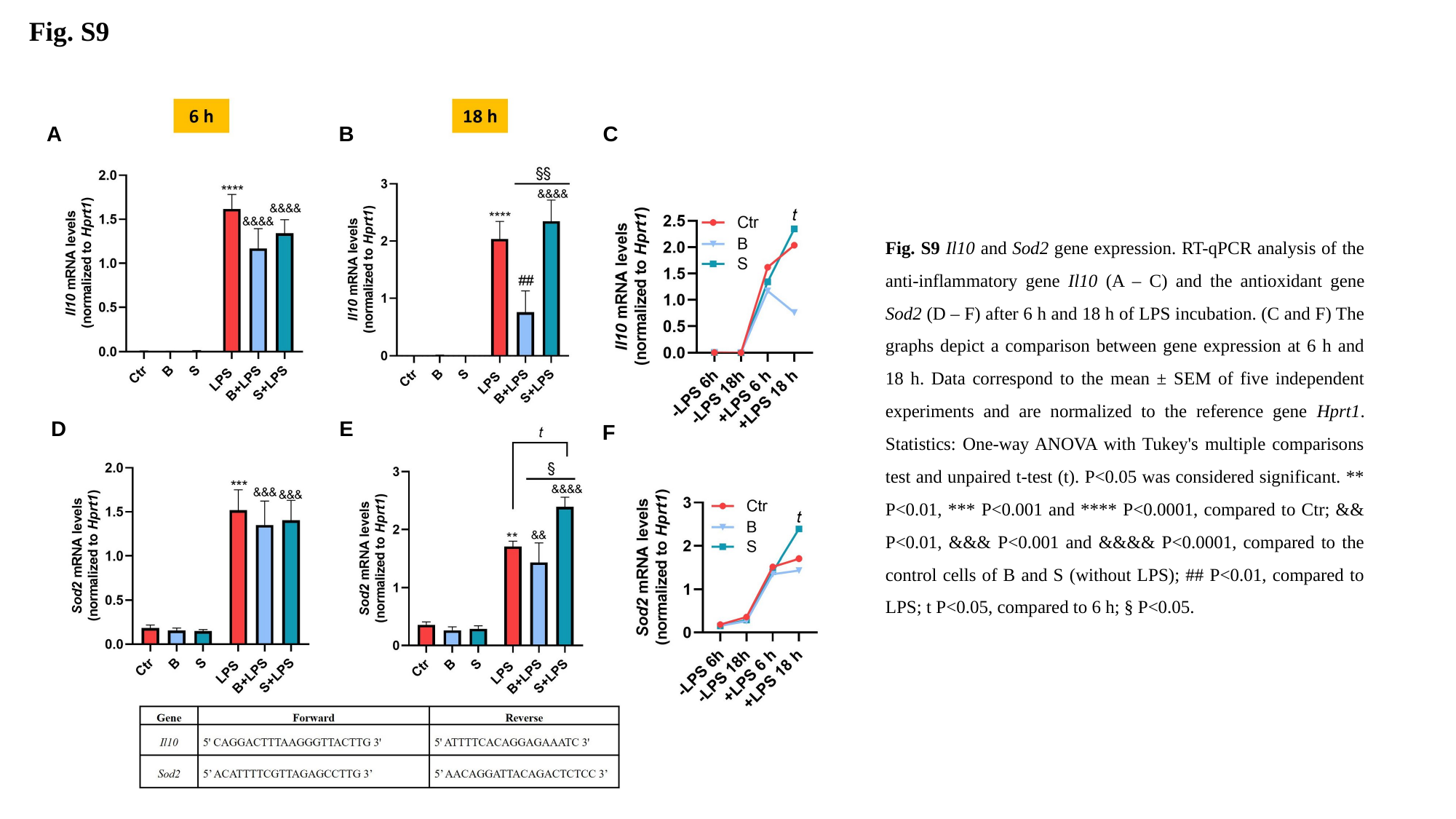

Fig. S9
A
B
C
Fig. S9 Il10 and Sod2 gene expression. RT-qPCR analysis of the anti-inflammatory gene Il10 (A – C) and the antioxidant gene Sod2 (D – F) after 6 h and 18 h of LPS incubation. (C and F) The graphs depict a comparison between gene expression at 6 h and 18 h. Data correspond to the mean ± SEM of five independent experiments and are normalized to the reference gene Hprt1. Statistics: One-way ANOVA with Tukey's multiple comparisons test and unpaired t-test (t). P<0.05 was considered significant. ** P<0.01, *** P<0.001 and **** P<0.0001, compared to Ctr; && P<0.01, &&& P<0.001 and &&&& P<0.0001, compared to the control cells of B and S (without LPS); ## P<0.01, compared to LPS; t P<0.05, compared to 6 h; § P<0.05.
D
E
F

## Slide 13
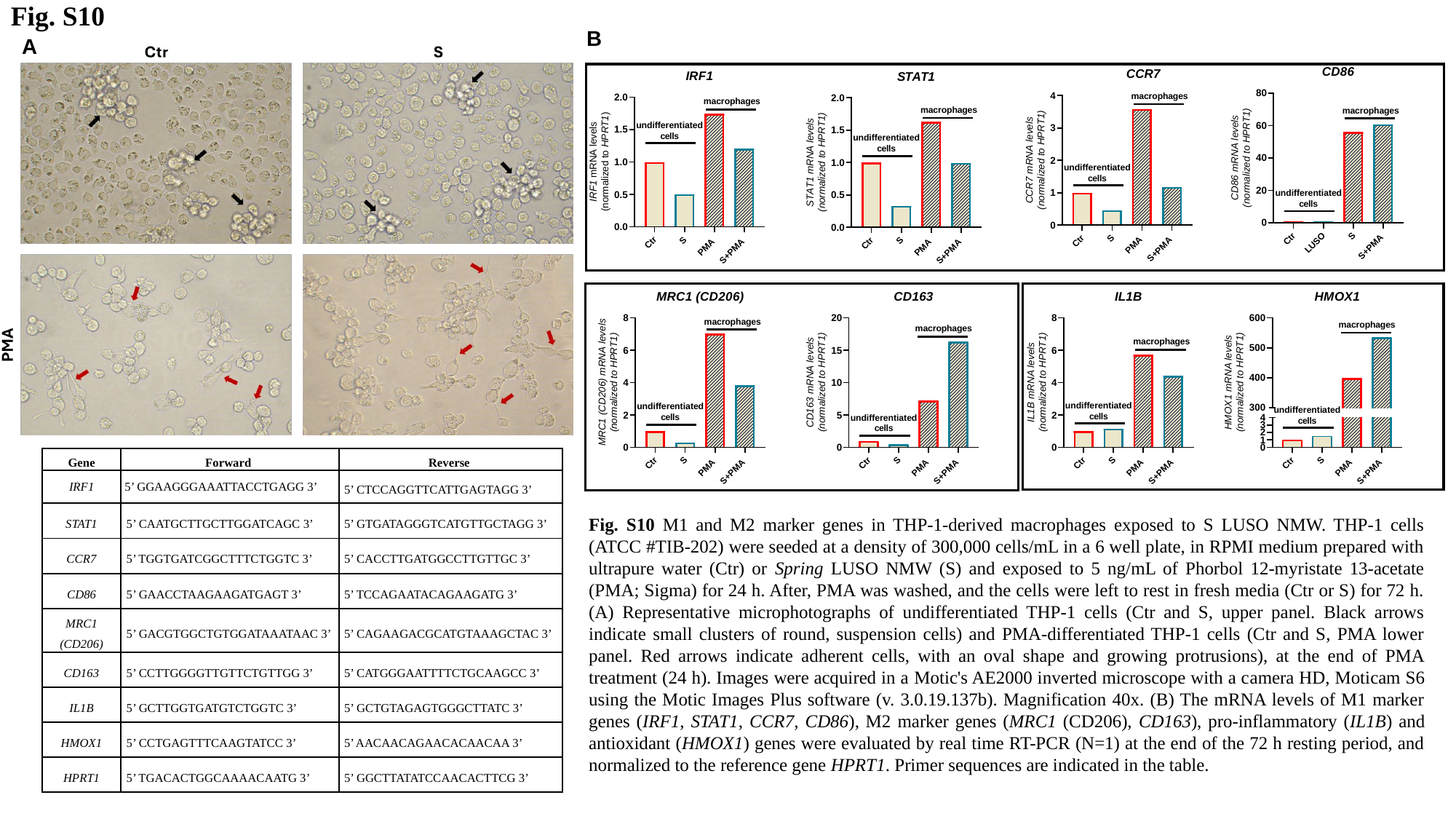

Fig. S10
B
A
| Gene | Forward | Reverse |
| --- | --- | --- |
| IRF1 | 5’ GGAAGGGAAATTACCTGAGG 3’ | 5’ CTCCAGGTTCATTGAGTAGG 3’ |
| STAT1 | 5’ CAATGCTTGCTTGGATCAGC 3’ | 5’ GTGATAGGGTCATGTTGCTAGG 3’ |
| CCR7 | 5’ TGGTGATCGGCTTTCTGGTC 3’ | 5’ CACCTTGATGGCCTTGTTGC 3’ |
| CD86 | 5’ GAACCTAAGAAGATGAGT 3’ | 5’ TCCAGAATACAGAAGATG 3’ |
| MRC1 (CD206) | 5’ GACGTGGCTGTGGATAAATAAC 3’ | 5’ CAGAAGACGCATGTAAAGCTAC 3’ |
| CD163 | 5’ CCTTGGGGTTGTTCTGTTGG 3’ | 5’ CATGGGAATTTTCTGCAAGCC 3’ |
| IL1B | 5’ GCTTGGTGATGTCTGGTC 3’ | 5’ GCTGTAGAGTGGGCTTATC 3’ |
| HMOX1 | 5’ CCTGAGTTTCAAGTATCC 3’ | 5’ AACAACAGAACACAACAA 3’ |
| HPRT1 | 5’ TGACACTGGCAAAACAATG 3’ | 5’ GGCTTATATCCAACACTTCG 3’ |
Fig. S10 M1 and M2 marker genes in THP-1-derived macrophages exposed to S LUSO NMW. THP-1 cells (ATCC #TIB-202) were seeded at a density of 300,000 cells/mL in a 6 well plate, in RPMI medium prepared with ultrapure water (Ctr) or Spring LUSO NMW (S) and exposed to 5 ng/mL of Phorbol 12-myristate 13-acetate (PMA; Sigma) for 24 h. After, PMA was washed, and the cells were left to rest in fresh media (Ctr or S) for 72 h. (A) Representative microphotographs of undifferentiated THP-1 cells (Ctr and S, upper panel. Black arrows indicate small clusters of round, suspension cells) and PMA-differentiated THP-1 cells (Ctr and S, PMA lower panel. Red arrows indicate adherent cells, with an oval shape and growing protrusions), at the end of PMA treatment (24 h). Images were acquired in a Motic's AE2000 inverted microscope with a camera HD, Moticam S6 using the Motic Images Plus software (v. 3.0.19.137b). Magnification 40x. (B) The mRNA levels of M1 marker genes (IRF1, STAT1, CCR7, CD86), M2 marker genes (MRC1 (CD206), CD163), pro-inflammatory (IL1B) and antioxidant (HMOX1) genes were evaluated by real time RT-PCR (N=1) at the end of the 72 h resting period, and normalized to the reference gene HPRT1. Primer sequences are indicated in the table.

## Slide 14
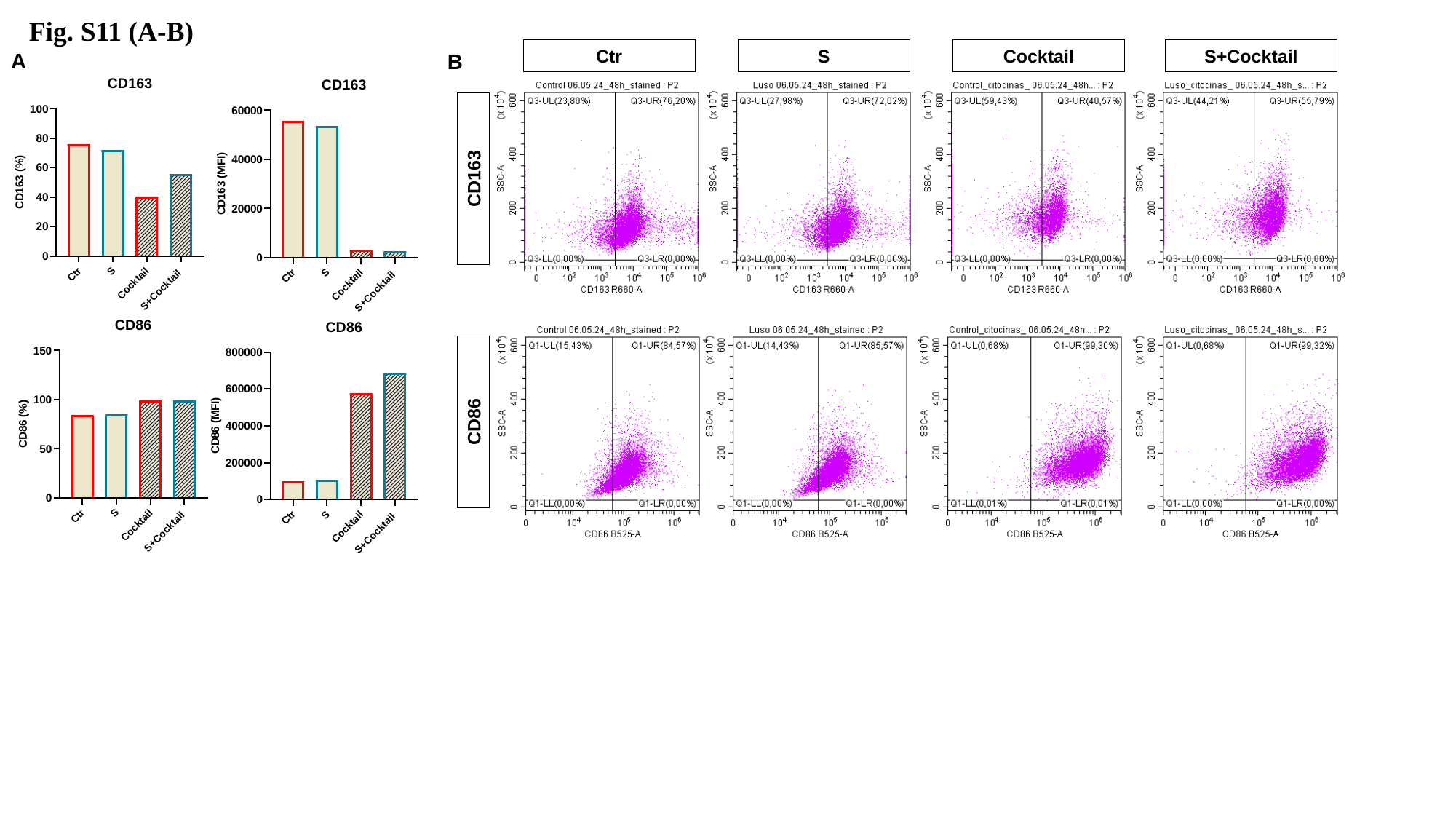

Fig. S11 (A-B)
A
B
Ctr
S
Cocktail
S+Cocktail
CD163
CD86

## Slide 15
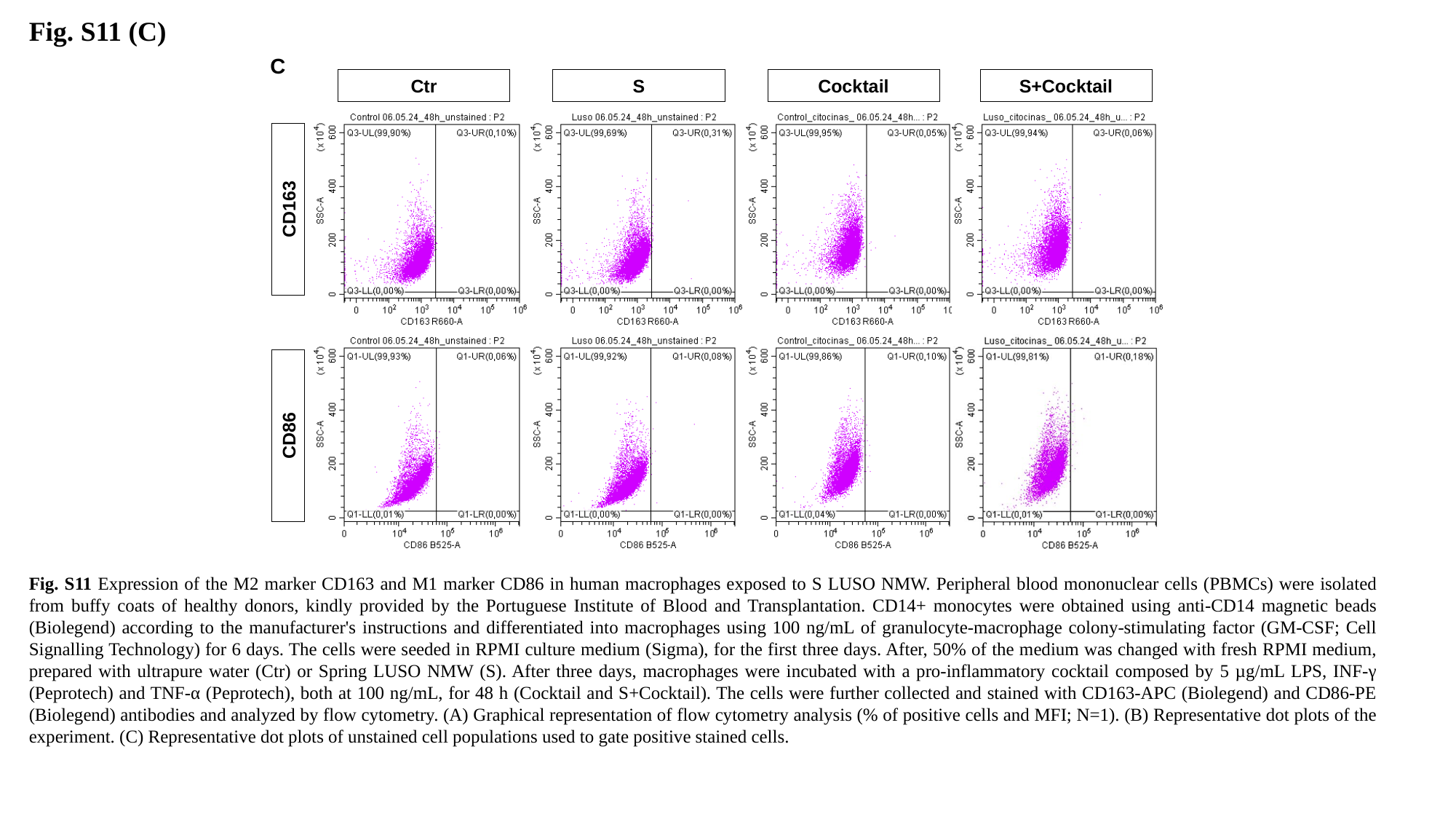

Fig. S11 (C)
C
Ctr
S
Cocktail
S+Cocktail
CD163
CD86
Fig. S11 Expression of the M2 marker CD163 and M1 marker CD86 in human macrophages exposed to S LUSO NMW. Peripheral blood mononuclear cells (PBMCs) were isolated from buffy coats of healthy donors, kindly provided by the Portuguese Institute of Blood and Transplantation. CD14+ monocytes were obtained using anti-CD14 magnetic beads (Biolegend) according to the manufacturer's instructions and differentiated into macrophages using 100 ng/mL of granulocyte-macrophage colony-stimulating factor (GM-CSF; Cell Signalling Technology) for 6 days. The cells were seeded in RPMI culture medium (Sigma), for the first three days. After, 50% of the medium was changed with fresh RPMI medium, prepared with ultrapure water (Ctr) or Spring LUSO NMW (S). After three days, macrophages were incubated with a pro-inflammatory cocktail composed by 5 µg/mL LPS, INF-γ (Peprotech) and TNF-α (Peprotech), both at 100 ng/mL, for 48 h (Cocktail and S+Cocktail). The cells were further collected and stained with CD163-APC (Biolegend) and CD86-PE (Biolegend) antibodies and analyzed by flow cytometry. (A) Graphical representation of flow cytometry analysis (% of positive cells and MFI; N=1). (B) Representative dot plots of the experiment. (C) Representative dot plots of unstained cell populations used to gate positive stained cells.

## Slide 16
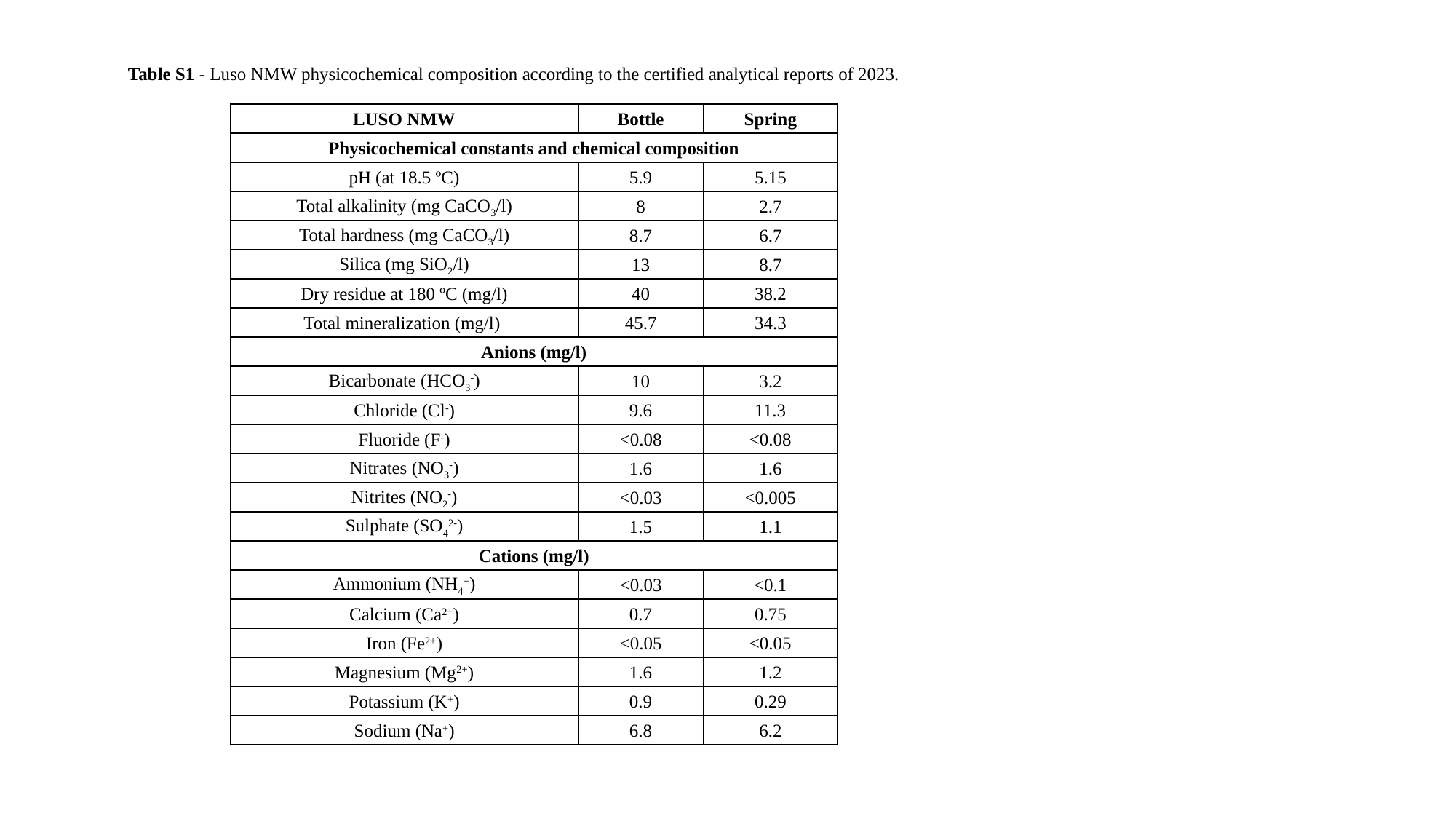

Table S1 - Luso NMW physicochemical composition according to the certified analytical reports of 2023.
| LUSO NMW | Bottle | Spring |
| --- | --- | --- |
| Physicochemical constants and chemical composition | | |
| pH (at 18.5 ºC) | 5.9 | 5.15 |
| Total alkalinity (mg CaCO3/l) | 8 | 2.7 |
| Total hardness (mg CaCO3/l) | 8.7 | 6.7 |
| Silica (mg SiO2/l) | 13 | 8.7 |
| Dry residue at 180 ºC (mg/l) | 40 | 38.2 |
| Total mineralization (mg/l) | 45.7 | 34.3 |
| Anions (mg/l) | | |
| Bicarbonate (HCO3-) | 10 | 3.2 |
| Chloride (Cl-) | 9.6 | 11.3 |
| Fluoride (F-) | <0.08 | <0.08 |
| Nitrates (NO3-) | 1.6 | 1.6 |
| Nitrites (NO2-) | <0.03 | <0.005 |
| Sulphate (SO42-) | 1.5 | 1.1 |
| Cations (mg/l) | | |
| Ammonium (NH4+) | <0.03 | <0.1 |
| Calcium (Ca2+) | 0.7 | 0.75 |
| Iron (Fe2+) | <0.05 | <0.05 |
| Magnesium (Mg2+) | 1.6 | 1.2 |
| Potassium (K+) | 0.9 | 0.29 |
| Sodium (Na+) | 6.8 | 6.2 |

## Slide 17
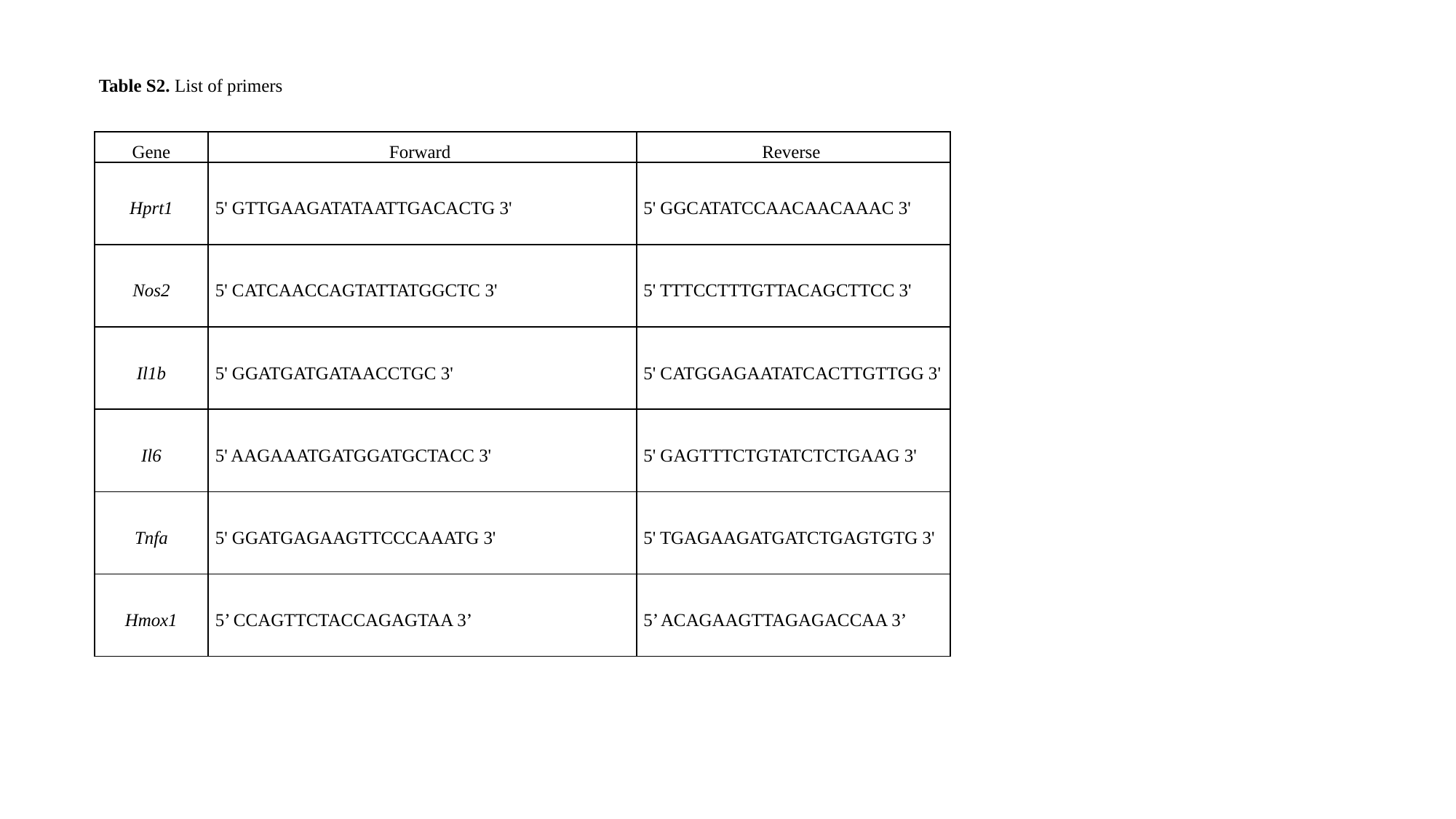

Table S2. List of primers
| Gene | Forward | Reverse |
| --- | --- | --- |
| Hprt1 | 5' GTTGAAGATATAATTGACACTG 3' | 5' GGCATATCCAACAACAAAC 3' |
| Nos2 | 5' CATCAACCAGTATTATGGCTC 3' | 5' TTTCCTTTGTTACAGCTTCC 3' |
| Il1b | 5' GGATGATGATAACCTGC 3' | 5' CATGGAGAATATCACTTGTTGG 3' |
| Il6 | 5' AAGAAATGATGGATGCTACC 3' | 5' GAGTTTCTGTATCTCTGAAG 3' |
| Tnfa | 5' GGATGAGAAGTTCCCAAATG 3' | 5' TGAGAAGATGATCTGAGTGTG 3' |
| Hmox1 | 5’ CCAGTTCTACCAGAGTAA 3’ | 5’ ACAGAAGTTAGAGACCAA 3’ |
